# Modeling neuroinflammatory interactions between microglia and astrocytes in a human iPSC-based coculture platform

**DOI:** 10.1186/s12964-025-02304-x

**Published:** 2025-06-20

**Authors:** Iisa Tujula, Tanja Hyvärinen, Johanna Lotila, Julia Rogal, Dimitrios Voulgaris, Lassi Sukki, Kaisa Tornberg, Katri Korpela, Henna Jäntti, Tarja Malm, Anna Herland, Pasi Kallio, Susanna Narkilahti, Sanna Hagman

**Affiliations:** 1 Neuroimmunology research group, Faculty of Medicine and Health Technology, Tampere University, Tampere, Finland; 2 Science for Life Laboratory, Division of Nanobiotechnology, Department of Protein Science, Royal Institute of Technology (KTH), Solna, 171 65 Sweden; 3 AIMES - Center for the Advancement of Integrated Medical and Engineering Sciences, Karolinska Institutet and KTH Royal Institute of Technology, Stockholm, Sweden; 4 Department of Neuroscience, Karolinska Institutet, Stockholm, SE-171 77 Sweden; 5 Micro- and Nanosystems Research Group, Faculty of Medicine and Health Technology, Tampere University, Tampere, Finland; 6 Neuroinflammation research group, Faculty of Health Sciences, A.I. Virtanen Institute for Molecular Sciences, University of Eastern Finland, Kuopio, Finland; 7 NeuroGroup, Faculty of Medicine and Health Technology, Tampere University, Tampere, Finland

**Keywords:** Astrocytes, Disease modeling, Glial crosstalk, iPSC, Microglia, Microphysiological system, Neuroinflammation

## Abstract

**Background:**

Microglia and astrocytes are central mediators of neuroinflammation in several neurodegenerative diseases. Their intricate crosstalk and contributions to pathogenesis remain elusive, highlighting the need for innovative in vitro approaches for investigating glial interactions in neuroinflammation. This study aimed to develop advanced human-based glial coculture models to explore the inflammatory interactions of microglia and astrocytes in vitro.

**Methods:**

Human induced pluripotent stem cell (iPSC)-derived microglia and astrocytes were cultured both in conventional culture dishes and in a microfluidic coculture platform. This platform features separate compartments for both cell types, enabling the creation of distinct microenvironments with spontaneous migration of microglia toward astrocytes through interconnecting microtunnels. To induce inflammatory activation, glial cultures were stimulated with lipopolysaccharide (LPS), a combination of tumor necrosis factor-α (TNF-α) and interleukin-1β (IL-1β), or interferon-γ (IFN-γ) for 24 h. Glial activation and interactions were analyzed with immunocytochemistry, the secretion of inflammatory factors from the culture media was measured, and microglial migration was quantified.

**Results:**

Microglia–astrocyte cocultures were generated in both conventional cultures and the microfluidic platform. Inflammatory stimulation with LPS and TNF-α/IL-1β elicited cell type-specific responses in microglia and astrocytes, respectively. LPS stimulation of cocultures induced lower secretion of several inflammatory mediators, suggesting dampening of microglial inflammatory responses when cocultured with astrocytes. Notably, inflammatory interaction between glial cells was demonstrated by increased level of IL-10 after TNF-α/IL-1β stimulation in cocultures compared with monocultures. The microfluidic coculture platform enabled the parallel study of microglial migration, glial activation and phagocytic function, thereby facilitating the investigation of glial responses within distinct inflammatory microenvironments. Furthermore, glial inflammatory responses and interactions were demonstrated in the controlled microenvironments of the microfluidic coculture platform. The inflammatory coculture environment was associated with elevated levels of complement component C3, emphasizing the intricate interplay between microglia and astrocytes.

**Conclusions:**

Our results depict an elaborate inflammatory interaction between iPSC-derived microglia and astrocytes via reciprocal molecular signaling. Importantly, the microfluidic coculture platform established in this study provides a more functional and advanced setup for investigating inflammatory glial interactions in vitro.

**Supplementary Information:**

The online version contains supplementary material available at 10.1186/s12964-025-02304-x.

## Background

Central nervous system (CNS)-resident glial cell types, microglia and astrocytes, are increasingly recognized as not only passive support cells but also active mediators of neuroinflammation in various neurodegenerative diseases, including Alzheimer’s disease (AD), Parkinson’s disease (PD), amyotrophic lateral sclerosis and multiple sclerosis (MS) [1, 2]. Though these diseases occur via distinct pathogenetic mechanisms, such as protein aggregation, autoimmune reactions or genetic mutations, they all exhibit chronic neuroinflammation as a common feature [3]. Microglia and astrocytes play pivotal roles in maintaining CNS homeostasis, supporting neuronal function, remodeling synapses and mediating inflammatory responses [2]. Upon CNS insult, they may adopt a diverse range of cellular states, resulting in morphological, molecular, and functional changes in response to pathological environmental cues [4, 5]. While the primary aim of glial cell activation is to resolve inflammation and promote tissue repair, sustained inflammatory reactions are harmful and promote neurodegeneration [2]. The context-specific molecular mechanisms that drive inflammatory activation and dynamic neuroimmune responses of microglia and astrocytes are currently the focus of extensive research [6–10].

Microglia and astrocytes do not function independently but modulate the functions of the other through tight bidirectional communication via soluble factors such as cytokines, chemokines, and growth factors [11, 12]. Microglia are highly motile, constantly monitoring their environment and providing initial responses to CNS insults [13]. While microglia are often placed as the primary drivers of neuroimmune responses, evidence suggests that astrocytes can also serve as a significant source of inflammatory mediators [14]. The importance of crosstalk between astrocytes and microglia in neurodegenerative contexts has been elegantly demonstrated in a previous study [15]. This study showed that a specific reactive astrocyte state was induced by the release of inflammatory mediators, including tumor necrosis factor (TNF)-α, interleukin (IL)-1α and the complement component C1q, from lipopolysaccharide (LPS)-activated microglia. These reactive astrocytes, identified by the upregulation of complement component 3 (C3), contribute to neuronal death and synapse loss in vitro and their presence has been confirmed in various neurodegenerative disorders [15]. However, the underlying mechanisms of this elaborate inflammatory crosstalk between glial cells and its contribution to the spatiotemporal progression of neuroinflammatory pathologies remain largely unclear.

Recent breakthroughs in human induced pluripotent stem cell (iPSC) differentiation technology have enabled the in vitro study of human microglia [6, 16, 17] and astrocytes [18–20], providing valuable insights into the underlying mechanisms of neuroinflammation and neurodegenerative diseases. The power of disease-specific iPSC models in understanding neuroinflammation was demonstrated in our recent study. By using iPSC-derived microglia from patients with MS, we showed significant transcriptional changes and altered cytokine release, indicating intrinsic immune activation state potentially contributing to the neuroinflammatory environment in MS [21]. In addition to monocultures, the neuroinflammatory functions of iPSC-derived glial cells have been studied with different coculture models [22]. Some studies have also been performed using more complex cell culture setups, such as 3D neural organoids or triculture of neurons, astrocytes and microglia, to investigate neuroinflammation in MS and AD [23–25]. Similarly, a range of stimuli, including cytokines, aggregated proteins and endotoxins, have been used to model specific inflammatory responses in microglia and astrocytes [22]. However, as the complexity of coculture models increases, so does the difficulty of identifying cell type-specific responses and certain cell–cell interactions [22, 26].

Conventional culture dishes offer limited control over culture design and cannot replicate the distinct microenvironments of the CNS [27]. To overcome these limitations, microfluidic technology has enabled the production of compartmentalized microphysiological systems and more advanced organ-on-a-chip models to study cellular functions with enhanced accuracy and physiological relevance [27]. We and others have utilized microfluidic platforms for various CNS applications. These include the isolation of neuronal axons and networks [28–32], the creation of pathologically relevant disease models, such as for AD and PD, by studying the effects of aggregated proteins [33–36], and the investigation of interactions between inflammatory glia and neuronal cells [34, 37, 38]. Previously, we demonstrated the effects of astrocytes on axonal growth in a neuroinflammatory environment using a microfluidic platform [37]. However, the interactions between microglia and astrocytes have not been widely investigated.

In the present study, our aim was to investigate the inflammatory responses of iPSC-derived microglia and astrocytes in monocultures (MG and AS), and their interaction in cocultures (MG/AS). Inflammatory stimuli elicited cell type-specific responses in glial cells, which were altered under coculture conditions, indicating reciprocal signaling between glial cells. In addition to studying the effects of several inflammatory stimuli in conventional cultures, we established a microfluidic coculture platform with astrocytes and microglia. The compartmentalized design enabled the creation of separate, interconnected inflammatory environments. It also enabled spontaneous microglial migration into the astrocyte compartment resulting in modulated immune responses driven by glial interaction. Finally, we observed an upregulation of C3 in microglia and astrocytes upon inflammatory stimulation and further potentiation in glial cocultures, suggesting that C3 contributes to glial crosstalk. Thus, the glial coculture models successfully mimic glial interactions and enhance our understanding of how microglia and astrocytes together contribute to neuroinflammation.

## Materials and methods

### Differentiation of iPSC-derived microglia

The human iPSC line UTA.04511.WTs [39] was used in this study for microglial differentiation. The line was derived and characterized at the Faculty of Medicine and Health Technology (MET), Tampere University, Finland. The iPSC line was expanded in feeder-free culture on 0.6 µg/cm^2^ recombinant human laminin-521 (LN521, BioLamina) in Essential8™ Flex media (E8 flex, Thermo Fisher Scientific), and passaged with TrypLE™ Select Enzyme and Defined Trypsin Inhibitor (DTI) (both from Thermo Fisher Scientific) in the presence of 10 µM Rock inhibitor (ROCKi, Y-27632, StemCell Technologies) as described previously [40]. The pluripotency of the iPSC line was monitored on a regular basis, and all cultures maintained normal karyotypes and were free of mycoplasma.

**Fig. 1:**
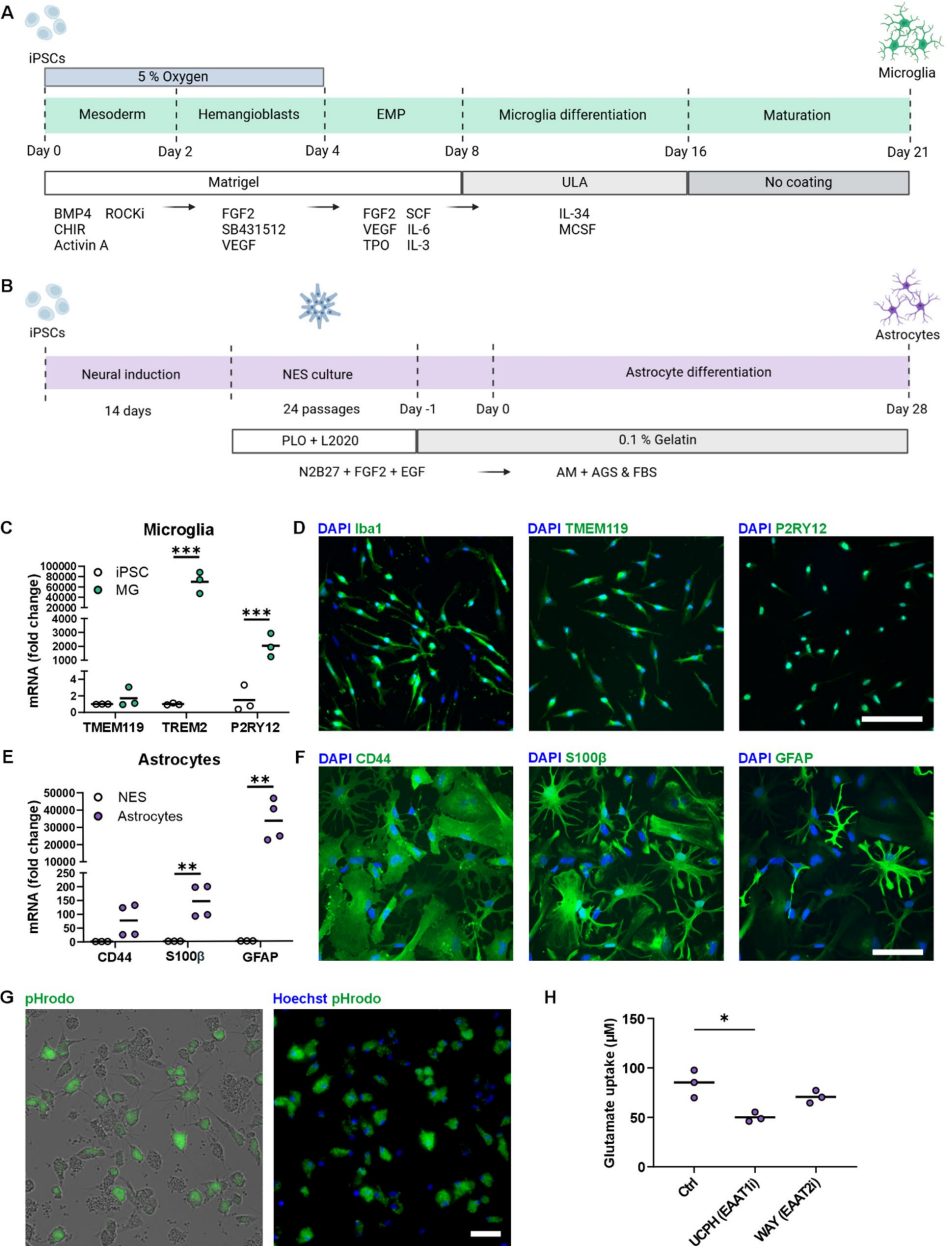
Characterization of differentiated human iPSC-derived microglia and astrocytes. **A, B** Schematic timelines of **(A)** microglia and **(B)** astrocyte differentiations. Created in BioRender. Hagman, S. (2025) https://BioRender.com/loiww5m. **C** RT–qPCR analysis of microglial expression of the genes *TMEM119*, *TREM2*, and *P2RY12* (*n* = 3, with 2 independent differentiations). **D** Representative immunofluorescence images of microglia characterized by the markers Iba1, TMEM119 and P2RY12 on Day 21. Scale bar is 100 μm. **E** RT–qPCR analysis of astrocyte expression of the genes *CD44*, *S100β* and *GFAP* (*n* = 3–4, with 2 independent differentiations). **F** Representative immunofluorescence images of astrocytes characterized by the markers CD44, S100β and GFAP on Day 31. Scale bar is 100 μm. **G** Representative live and fluorescence images of microglial phagocytosis of fluorescent pHrodo zymosan bioparticles. Scale bar is 50 μm. **H** Astrocyte glutamate uptake under control conditions and after inhibitor treatment (UCPH & WAY) (*n* = 3). The data are presented as the means with individual values. *p < 0.05, **p < 0.01, ***p < 0.001; Student’s t test

The in-house-produced microglia were differentiated according to a previous publication [17] with minor modifications (Fig. 1A). Briefly, on microglial differentiation Day 0, iPSCs were plated on Matrigel (Corning)-coated dishes at 9 000–20 0000 cells/cm^2^ and cultured under hypoxic conditions (5% O2, 5% CO2, 37 °C) until Day 4. On Days 0 and 1, the cells were cultured in E8 flex media supplemented with 5 ng/ml BMP4, 25 ng/ml activin A (both from Peprotech), 1 µM CHIR 99,021 (Axon) and ROCKi (Day 0: 10 µM and Day 1: 1 µM). During Days 2 to 8, the cells were cultured in base media containing DMEM/F-12 without glutamine, 1X GlutaMAX, 543 mg/L sodium bicarbonate (all from Thermo Fisher Scientific), 14 µg/L sodium selenite, 64 mg/L L-ascorbic acid (both from Sigma–Aldrich) and 0.5% penicillin/streptomycin (P/S). On Days 2 and 3, the base media was supplemented with 100 ng/ml FGF2, 50 ng/ml VEGF (both from Peprotech), 10 µM SB431542 and 5 µg/ml insulin (both from Sigma–Aldrich). On Day 4, the cells were transferred to a normoxic incubator (5% CO2, 37 °C). From Day 4 until Day 8, the cells were cultured in base media supplemented with 50 ng/ml FGF2, 50 ng/ml VEGF, 50 ng/ml TPO, 50 ng/ml IL-6, 10 ng/ml SCF, 10 ng/ml IL-3 (all from Peprotech) and 5 µg/ml insulin (Sigma–Aldrich), and media changes were conducted daily. On Day 8, the floating erythromyeloid progenitor cells (EMPs) were collected and seeded at 64 000 cells/cm^2^ in ultralow attachment (ULA) dishes (Corning) in base media containing Iscove′s modified Dulbecco′s medium (IMDM, Thermo Fisher Scientific), 10% heat-inactivated fetal bovine serum (FBS, Sigma–Aldrich) and 0.5% P/S supplemented with 5 ng/ml MCSF, 100 ng/ml IL-34 (both from Peprotech) and 5 µg/ml insulin. From Day 10 onward, the cells were cultured in primitive macrophage (PM) media containing IMDM supplemented with 10% FBS (Sigma–Aldrich), 0.5% P/S, 10 ng/ml MCSF and 10 ng/ml IL-34 (both from Peprotech). The media was changed every other day until final plating for the experiments was performed on Day 16. From Day 16 onward, half of the media was changed daily. For characterization, microglia were seeded at 45 00 cells/cm^2^ in 96-well plates (PerkinElmer) and cultured for 5 days prior to fixation for immunocytochemistry. For RNA sample collection, the cells were plated at 42 00 cells/cm^2^ in 6-well plates (Thermo Fisher Scientific) and cultured for 6 days.

### Differentiation of iPSC-derived astrocytes

Astrocyte differentiation in this study was conducted utilizing neuroepithelial stem (NES) cell line control 7 (NESC7) [41], which was originally provided by the iPS Core Facility of Karolinska Institutet, Sweden. NESC7 cells were cultured and differentiated into astrocytes as described in a previous publication with minor modifications (Fig. 1B) [20]. Briefly, NESC7 cells were cultured in N2B27 media consisting of DMEM/F-12 GlutaMAX (Thermo Fisher Scientific), 1% N2 (Thermo Fisher Scientific), 0.1% B27 (Thermo Fisher Scientific) and 0.1% P/S supplemented with 10 ng/ml FGF2 (R&D Systems) and 10 ng/ml EGF (Sigma–Aldrich). The cells were cultured in culture flasks (T25 & T75, VWR) double-coated with 20 µg/ml poly-L-ornithine (PLO, Sigma–Aldrich) and 1:500 diluted murine Engelbreth-Holm-Swarm sarcoma derived laminin (L2020, Sigma–Aldrich). After passage 23–24, the cells were cultured in N2B27 media supplemented with a lower concentration of EGF (1 ng/ml). Full media change was conducted every other day, and on the days between media changes, growth factors were added to the culture media. The cells were passaged every 3–4 days at a ratio of 1:4–1:5 or at a density of 20 00–30 000 cells/cm^2^. Passaging was conducted using TrypLE™ Select Enzyme and DTI.

On Day − 1 of astrocyte differentiation, the NES cells were passaged and seeded in 6-well plates coated with 0.1% gelatin (Thermo Fisher Scientific) at 30 000 cells/cm^2^ in N2B27 media supplemented with 10 ng/ml FGF2 and 1 ng/ml EGF. On Day 0, the cells were washed once with phosphate-buffered saline (PBS) before the addition of astrocyte media (AM) (ScienCell) supplemented with 2% FBS and 1% AGS (both from ScienCell) and 0.1% P/S. Media change with AM media was conducted every other day throughout the differentiation period. The cells were cultured without passaging until Day 6, after which the following passages were conducted within 1–2 days upon reaching 95% confluency. Passaging was conducted using TrypLE™ Select Enzyme and DTI. The cells were seeded at 30 000 cells/cm^2^ for all passages, and after the second passage, the cells were cultured in cell culture flasks (T25 and T75). On Day 28, differentiated astrocytes were plated for experiments. For characterization, astrocytes were plated at 30 000 cells/cm^2^ in 96-well plates (for immunocytochemistry) or 24-well plates (for RNA sample collection) (both from Thermo Fisher Scientific). The cells were cultured for 3 days before fixation for immunocytochemical staining and for 4 days before RNA samples were collected.

### Establishment of microglia and astrocyte monocultures and cocultures

Microglia monocultures were prepared by seeding the cells in 96-well plates (PerkinElmer) at a density of 45 000 cells/cm^2^ (15 000 cells/well) on microglial differentiation Day 16 (Fig. 1A). The cells were allowed to mature for 5 days before the experiments were started. Half media changes with PM media were conducted daily.

Astrocyte monocultures were prepared by seeding the cells in PLO and LN521 double-coated 96-well plates at a density of 25 000 cells/cm^2^ (8 000 cells/well). Briefly, the well plates were coated with 0.1 mg/ml PLO for 1 h at 37 °C, after which they were washed three times with sterile water and air-dried at room temperature (RT). The well plates were subsequently coated with 15 µg/ml LN521 overnight at 4 °C. Astrocytes were plated in AM media. The media was changed to AM media supplemented with the microglia growth factors 10 ng/ml MCSF and IL-34 (AM++) on the day after plating. Astrocytes were cultured for 7 days before the experiments were started. Half media changes with AM + + were conducted daily.

Microglia and astrocyte cocultures were prepared in PLO and LN521 double-coated 96-well plates (coated as described above). First, astrocytes were seeded in well plates at a density of 25 000 cells/cm^2^ (8 000 cells/well) in AM media on Day − 7. A full media change and switch to AM + + was conducted on the day after plating. On Day − 5, microglia were plated on top of the astrocytes at a density of 45 000 cells/cm^2^ (15 000 cells/well) on microglial differentiation Day 16. The cells were cultured together in AM + + media for 5 days before the experiments were started on Day 0. Half of the media was changed daily.

### Design and fabrication of the microfluidic platform

An in-house-engineered microfluidic platform was used to culture microglia and astrocytes in their designated, interconnected compartments [30, 42]. The utilized microfluidic platform consists of two polydimethylsiloxane (PDMS) parts: a cell culture part and a medium reservoir part (Supplementary Fig. 1). The cell culture part has three separate cell compartments that are interconnected by microtunnels. There are two microglia compartments on the sides (length = 3 mm, width = 4 mm) that are connected to the microglia–astrocyte compartment in the middle (length = 5 mm, width = 4 mm) with 40 microtunnels (length = 250 μm, width = 10 μm, height = 3.5 μm). The microtunnels allow microglial migration between the compartments. The medium reservoir part consists of three separate media chambers, enabling near-complete fluidic isolation between the cell compartments and the utilization of cell type-specific culture media.

The microfluidic platform was fabricated from PDMS (SYLGARD 184, Dow Corning), and the cell compartments and microtunnels were treated with polyvinylpyrrolidone (PVP) (Sigma–Aldrich) [42]. The fabrication was conducted as described in a previous publication [43] with the following modifications. The fabricated molds were treated with trichloro(1 H,1 H,2 H,2 H-perfluorooctyl)silane (Sigma–Aldrich) to improve the demolding process. The 4 mm thick PDMS sheets for the medium chambers were covered with a thin layer of dish soap before laser cutting to wash away the cut debris from the surfaces.

### Assembly of the microfluidic platform and cell culture

The assembly of the microfluidic platform was conducted as described previously [30]. Briefly, HCl-cleaned ∅24 mm glass coverslips were coated with 0.25 mg/ml PLO at 37 °C for 1.5 h. Subsequently, the coverslips were washed three times with sterile H_2_O, allowed to air-dry at RT, and stored at 4 °C. The PVP-coated microfluidic platforms were sterilized by immersion in 70% ethanol and air-dried at RT prior to assembly. Thereafter, the microfluidic platforms were manually attached to the PLO-coated glass coverslips and the cell compartments were coated with 30 µg/ml LN521 overnight at 4 °C.

Microglia were seeded in their designated side compartments at a density of 25 000 cells/cm^2^ (3 000 cells/compartment) on microglial differentiation Day 16 (Day − 7 of the experimental setup) and 5 days prior to the seeding of astrocytes. The experimental setup deviated from conventional cocultures to allow microglial maturation while preventing excessive migration toward astrocytes before starting the experiments. During this period, PM media was used for the microglia compartments, and AM media was used for the middle compartment. On Day − 2 of the experiment, astrocytes were seeded into the middle compartment at a density of 45 000 cells/cm^2^ (9 000 cells/compartment) in AM + + media supplemented with microglial growth factors. The cells were cultured together in the microfluidic coculture platform for two days before the experiments were started on Day 0. Fresh media for the media chambers was changed daily throughout the culture without disturbing the cell compartments. Astrocytes and microglia were additionally cultured alone in the microfluidic platform to study inflammatory secretion and microglial migration, respectively. Cell seeding and culturing were conducted as in the cocultures, with the exception of utilizing cell type-specific media (PM or AM++) for all three compartments.

### Fluidic isolation in the microfluidic platform

To test fluidic isolation between the cell compartments in the microfluidic platform, FITC-conjugated dextran (15–25 kDa) (TdB Consultancy) was used. The dextran conjugates (50 µM) were added to the middle compartment, and their diffusion was evaluated for 24 h at 37 °C. The diffusion of the dextran into the side compartments was visualized with an Olympus IX51 microscope equipped with an Olympus DP30BW camera (Olympus Corporation). To quantify the diffusion of dextran between the compartments after 24 h, the absorbance of the medium samples at 490 nm was measured using a NanoDrop 1000 spectrophotometer (Thermo Fisher Scientific).

### Stimulation of inflammatory activation

Microglia and astrocyte monocultures and microglia and astrocyte cocultures were stimulated for 24 h using three different stimuli: 100 ng/ml LPS (Sigma–Aldrich), a combination of 10 ng/ml TNF-α/IL-1β (both from Peprotech), or 20 ng/ml interferon (IFN)-γ (Peprotech) in respective culture media (PM or AM++). After 24 h of treatment, viability staining was performed, the media were collected for secretion analysis, and the cultures were fixed for immunocytochemical staining.

The stimulation of inflammatory activation in the microfluidic coculture platform was conducted in a similar manner. On Day 0, culture media (PM or AM++) containing 100 ng/ml LPS, a combination of 10 ng/ml TNF-α/ IL-1β, or 20 ng/ml IFN-γ was added to each cell compartment and media chamber. For the migration studies, the cells were stimulated with 100 µM adenosine diphosphate (ADP) (Sigma–Aldrich). The cells were treated for 24 h after which culture media was collected from media chambers of both the microglia and astrocyte compartments. Additionally, one experiment was performed with selective stimulation of the middle compartment containing both microglia and astrocytes with a combination of 10 ng/ml TNF-α/ IL-1β or 20 ng/ml IFN-γ for 24 h and 7 d. Media from microglia compartments of the same microfluidic platform were pooled, but each microfluidic platform was collected separately. The cells were fixed for immunocytochemical staining.

### Immunocytochemistry

Immunocytochemistry (ICC) of conventional cultures was performed by first fixing the cultures for 15 min with 4% paraformaldehyde in PBS. The fixed samples were then blocked for 45 min with 10% normal donkey serum (NDS), 0.1% Triton X-100 and 1% bovine serum albumin (BSA) in PBS at RT. Primary antibody solutions were diluted in 1% NDS, 0.1% Triton X-100 and 1% BSA in PBS and incubated overnight at 4 °C. The secondary antibodies were diluted in 1% BSA in PBS and incubated for 1 h at RT. The samples were then mounted with ProLong™ Gold Antifade Mountant with DAPI (Thermo Fisher Scientific). ICC on microfluidic platforms was performed as described in a previous publication [30]. As a minor modification, the microfluidic platforms were washed three times with DAPI diluted 1:5 000 in PBS after secondary antibody incubation. The primary and secondary antibodies used are listed in Supplementary Table 1. Images were acquired with an Olympus IX51 inverted fluorescence microscope equipped with a Hamamatsu ORCA-Flash4.0 LT + sCMOS camera (Olympus Corporation), a DMi8 inverted microscope (Leica) and an LSM780 laser-scanning confocal microscope equipped with a Quasar spectral GaAsP detector (all from Carl Zeiss). Image analysis was performed with CellProfiler and CellProfiler Analyst softwares [44].

### RNA isolation and quantitative PCR

RNA was extracted from astrocytes, microglia, NES cells and iPSCs utilizing a NucleoSpin RNA kit (Macherey-Nagel). The purity and concentration of RNA were measured with a NanoDrop 1000 (Thermo Fisher Scientific). The RNA was converted to cDNA with a High Capacity cDNA Reverse Transcription Kit (Thermo Fisher Scientific). The gene expression levels of the astrocytic markers *CD44* (Hs01075864_m1), *S100β* (Hs00902901_m1) and *GFAP* (Hs00909236_m1) and the microglial markers *TMEM119* (Hs01938722_u1), *P2RY12* (Hs00224470_m1) and *TREM2* (Hs00219132_m1) were analyzed with TaqMan assays via the ABI QuantStudio 12 K Flex Real-Time PCR System (Thermo Fisher Scientific). The samples were run in triplicate. The data were analyzed via the ΔΔCt method. Astrocyte data were normalized to those of the housekeeping gene *GUSB* (Hs00939627_m1) and NES samples. The microglia results were normalized to housekeeping gene *GAPDH* (Hs99999905_ m1) and the iPSC sample.

### Glutamate uptake assay

Glutamate uptake by differentiated astrocytes was analyzed with a glutamate assay kit (Abcam). Astrocytes were plated at 100 000 cells/cm^2^ in a 24-well plate (Thermo Fisher Scientific) and cultured for 4 days in AM medium before the assay was started. First, the cells were washed twice with PBS (with Ca^++^ and Mg^++^), followed by a 30 min incubation at 37 °C with 1:10 diluted dimethyl sulfoxide (DMSO, control, Sigma–Aldrich), 1.5 mM UCPH-101 (EAAT-1 inhibitor, Abcam), or 1 mM WAY213613 (EAAT-2 inhibitor, Tocris) in Hank’s balanced salt solution (with Ca^++^ and Mg^++^) (HBSS, Gibco). After incubation, HBSS containing 100 µM glutamate was added to the cells, which were subsequently incubated for 60 min at 37 °C. The cells were then washed three times with PBS and lysed with assay buffer. The concentration of glutamate in the cell lysates was analyzed following the manufacturer’s protocol and each sample was run in duplicate.

### Mesoscale and ELISA

The secretion of cytokines and chemokines (TNF-α, IL-1β, IL-6, GM-CSF, IL-10, CCL2, CXCL5, CXCL8, and CXCL10) from the collected culture medium was measured using a U-PLEX Custom Biomarker Group 1 (human) Assay (Meso Scale Diagnostics) according to the manufacturer’s protocol. Secretion of C3 was measured using an R-PLEX Human Complement C3 Assay (Meso Scale Diagnostics). FBS containing media was utilized as a negative control for both assays. The plates were run using a MESO QuickPlex SQ 120 instrument and the results were analyzed with DISCOVERY WORKBENCH^®^ software (v4.0) (Meso Scale Diagnostics). If the measured values were below the detection limit, the values were set to 0. Values above the maximum detection limit were set to the maximum detectable value. A human IL-6 uncoated ELISA kit (Thermo Fisher Scientific) was used to measure the levels of IL-6 in the medium after selective middle microglia–astrocyte compartment inflammatory stimulation with TNF-α/IL-1β. The absorbance was measured at 450 nm (Wallac Victor 1420). All the samples were run in duplicates.

### Phagocytosis assay

The phagocytic capacity of microglia was investigated using pHrodo™ Green Zymosan Bioparticles™ Conjugate for Phagocytosis (P35365, Thermo Fisher Scientific). pHrodo bioparticles were added to cells at 100 µg/ml in Opti-MEM (Thermo Fisher Scientific) supplemented with the microglial growth factors IL-34 and MCSF (both 10 ng/ml). After 6 h of incubation, the nuclei were stained with Hoechst 33,342 (1:1 000, Thermo Fisher Scientific), and the cells were imaged immediately with an Olympus IX51 fluorescence microscope equipped with a Hamamatsu ORCA-Flash4.0 LT + sCMOS camera.

### Viability staining

The viability of the cultured cells after inflammatory stimulation was analyzed via a viability/cytotoxicity kit for mammalian cells (Thermo Fisher Scientific). The cultures were incubated for 30 min at 37 °C with green fluorescent calcein-AM (0.5 µM) for the detection of live cells and red fluorescent ethidium homodimer-1 (EthD-1) (0.5 µM) for the detection of dead cells. The samples were imaged with an Olympus IX51 microscope equipped with a Hamamatsu ORCA-Flash4.0 LT + sCMOS camera.

### Statistical analysis

The normality of the data was determined with the Shapiro–Wilk test. Statistical analysis of normally distributed data was performed with Student’s t test or one-way ANOVA with Tukey’s post hoc comparison. The nonparametric Mann–Whitney U test was used for nonnormally distributed data. A p value < 0.05 was considered statistically significant. Statistical significance is denoted as * *p* < 0.05, ** *p* < 0.01, and *** *p* < 0.001. All the statistical tests were performed with IMB SPSS Statistics software (version 29.0.1.0).

## Results

### Generation of cocultures of human iPSC-derived microglia and astrocytes exhibiting cell type-specific markers and functions

We used well-established published protocols to differentiate human iPSC-derived microglia [17] and astrocytes [20] (Fig. 1A and B). Successful microglial differentiation was confirmed by the increased gene expression of the typical markers *TREM2*, and *P2RY12* (Fig. 1C) and positive immunocytochemical staining for Iba1, TMEM119, and P2RY12 (Fig. 1D). Differentiated astrocytes, in turn, showed increased gene expression of the astrocyte-specific markers S100β and GFAP (Fig. 1E) and positive immunocytochemical staining for CD44, S100β and GFAP (Fig. 1F). Both cell types performed key functions: microglia exhibited phagocytosis activity through the uptake of pHrodo zymosan bioparticles (Fig. 1G), and astrocytes showed glutamate uptake. This was primarily mediated by the glutamate transporter EAAT1, as shown by significantly reduced uptake after treatment with the inhibitor UCPH-101 (Fig. 1H). Therefore, both microglia and astrocytes displayed cell type-specific characteristics and performed essential functions.

**Fig. 2:**
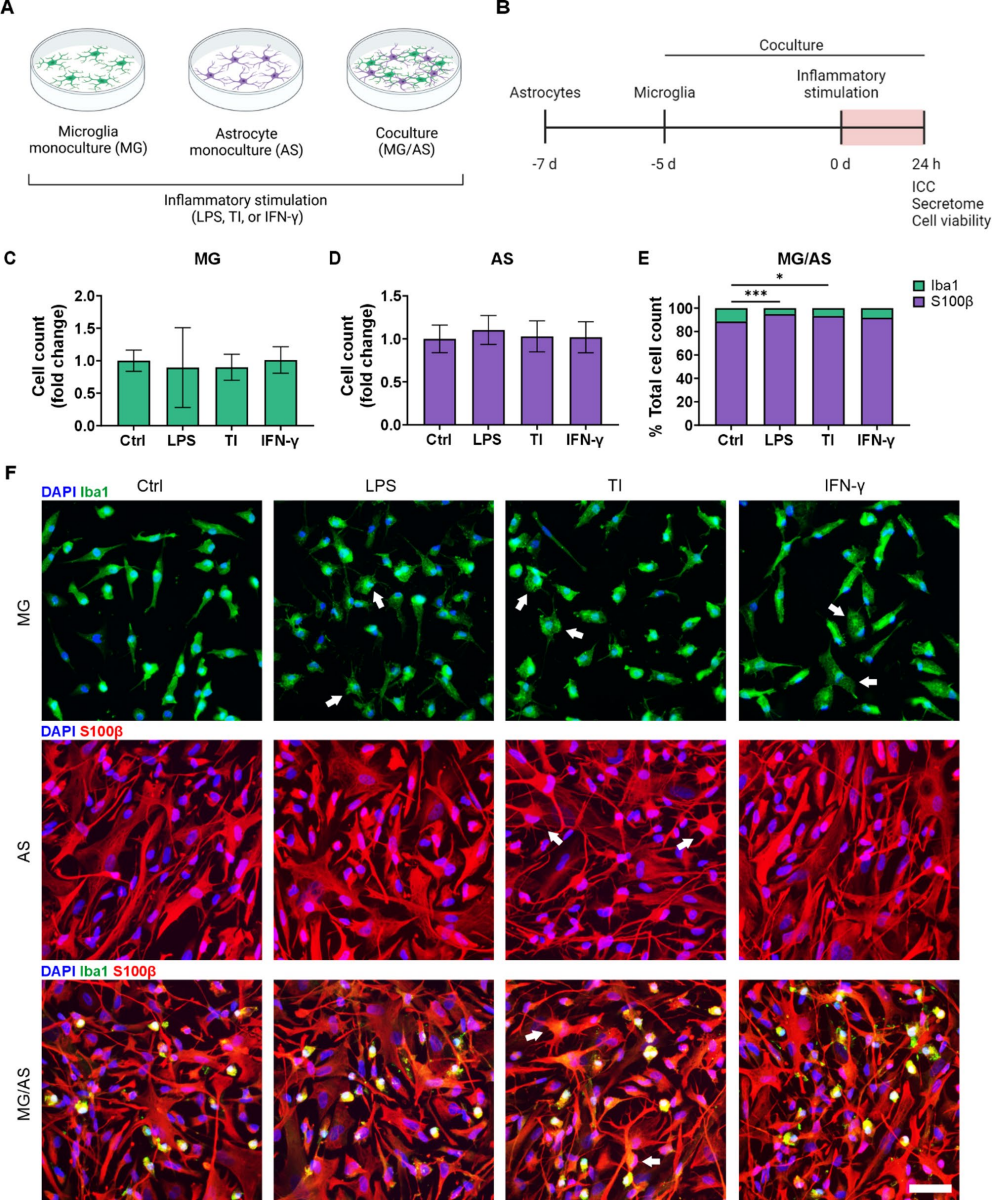
Neuroinflammatory stimulation induced morphological changes in generated microglia and astrocyte monocultures and cocultures. **A, B** Schematic figures of **(A)** the experimental setup and **(B)** timeline. Created in BioRender. Hagman, S. (2025) https://BioRender.com/loiww5m. **C, D** Quantified cell amounts of Iba1-positive microglia **(C)** and S100b-positive astrocytes **(D)** in monocultures are presented as the fold change (*n* = 18 images per condition; images from 2 experiments). The data are presented as the means ± SDs; *p* > 0.05. **E** Quantification of Iba1- and S100β-positive cells in cocultures presented as a percentage of the total cell count (*n* = 18 images per condition; images from 2 experiments). The data are presented as the means. **F** Representative immunofluorescence images showing observable morphological changes indicated by white arrows following inflammatory activation in both monocultures and cocultures. Scale bar is 50 μm. *p < 0.05, **p < 0.01, ***p < 0.001; one-way ANOVA with Tukey’s post hoc comparison. TI: TNF-α/IL-1β

To study the immune responses of both cell types, we prepared MG and AS monocultures and MG/AS cocultures (Fig. 2A and B). Equal amounts of microglia and astrocytes were plated for both monocultures and cocultures to enable their comparison. Quantification revealed that after six days, the stabilized cocultures consisted of 11% microglia and 89% astrocytes (Fig. 2E). Viability staining with calcein-AM and EthD-1 demonstrated high viability of the established cultures, which was also indicated by negative immunocytochemical staining for the apoptotic marker cleaved caspase-3 (Supplementary Fig. 2).

### Neuroinflammatory stimulation induces characteristic morphological changes without affecting cell viability

To study the immune responses of microglia and astrocytes, we stimulated the established cultures for 24 h with the following inflammatory stimuli: LPS, TNF-α/IL-1β or IFN-γ (Fig. 2A and B). First, we investigated whether stimulation had an effect on the overall number of cells and their proliferation. MG and AS monocultures presented no significant alterations in cell numbers following stimulation (Fig. 2C and D). The proportion of microglia in cocultures was more variable after stimulation and decreased with both LPS (5% vs. 11%, *p* < 0.001) and TNF-α/IL-1β stimulation (7% vs. 11%, *p* < 0.05) compared with the control conditions (Fig. 2E). Immunocytochemical staining for the proliferation marker Ki-67 revealed reduced proliferation in all the stimulation groups in the MG monocultures compared with the control (*p* < 0.001), which could partially explain a slight reduction in cell numbers (Supplementary Fig. 3).

We further investigated the potential effects of inflammatory stimulation on the morphology and viability of the prepared cultures. Observation of cell morphology revealed that all stimuli increased hypertrophy in MG monocultures, indicating transition to an activated state (Fig. 2F). TNF-α/IL-1β stimulation induced increased ramification in AS monocultures, whereas LPS or IFN-γ stimulation did not have observable effects. While astrocytes in cocultures demonstrated comparable morphological changes in response to TNF-α/IL-1β stimulation, microglial morphology was already more amoeboid under control conditions. Consequently, they did not demonstrate similar observable morphological changes as those in monocultures. Finally, quantification of viability staining with calcein-AM and EthD-1 confirmed high viability in all stimulated cultures (Supplementary Fig. 4), which was also supported by negative immunocytochemical staining for the apoptotic marker cleaved caspase-3 (Supplementary Fig. 5). In summary, inflammatory stimulation of glial cultures induced typical observable morphological changes in both microglia and astrocytes, indicating inflammatory activation with no detrimental effects on cell viability.

### Stimulation with LPS and TNF-α/IL-1β induces cell type-specific responses in microglia and astrocytes and triggers glial interaction in cocultures

**Fig. 3:**
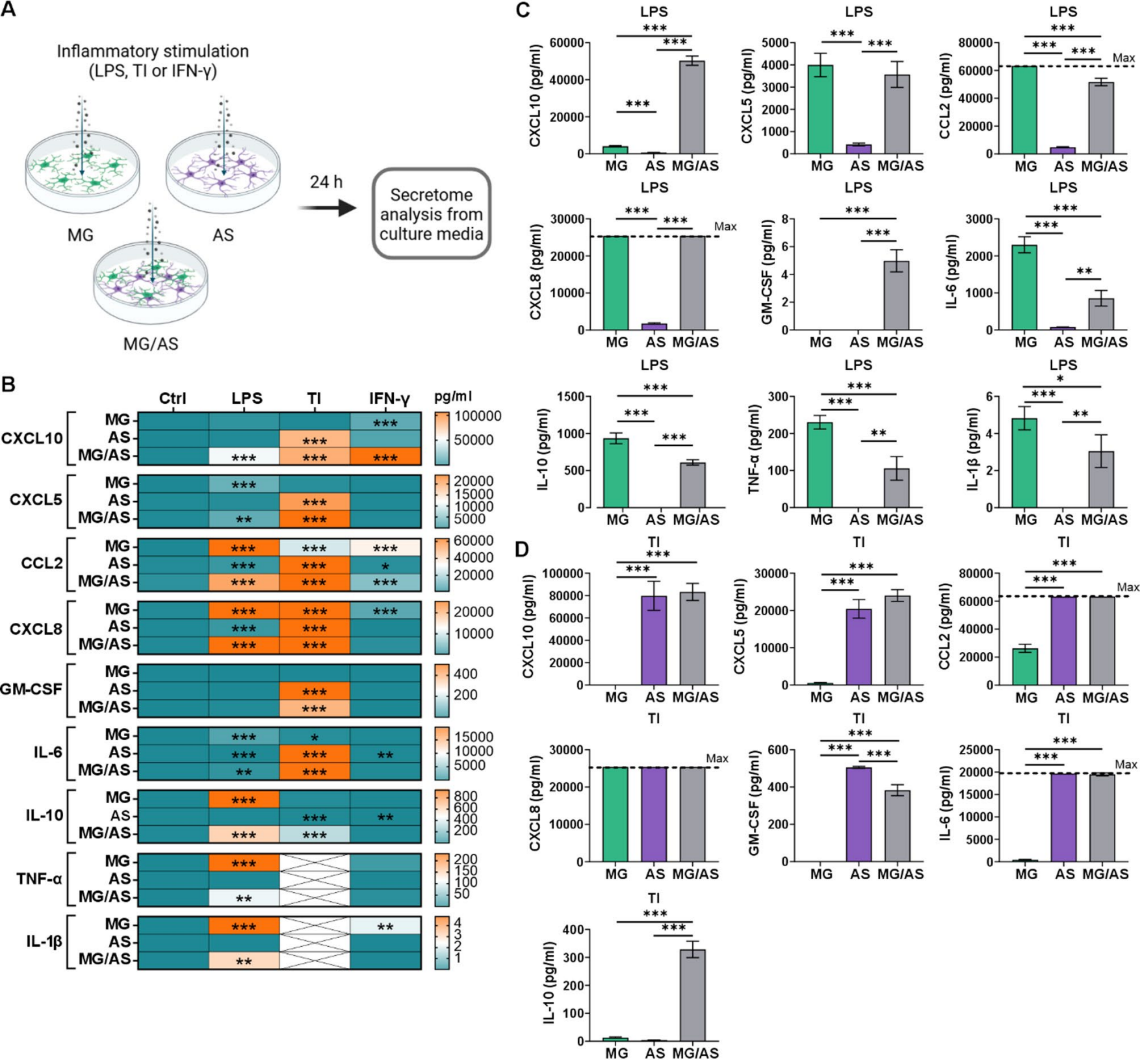
Inflammatory stimulation induces cell type-specific responses in microglia and astrocytes and altered responses in cocultures. **A** Schematic figure of inflammatory stimulation. Created in BioRender. Hagman, S. (2025) https://BioRender.com/loiww5m. **B** Heatmap of the secretion of inflammatory cytokines and chemokines in conventional cultures after 24 h of stimulation. Asterisks indicate statistical significance in comparison with the control (*n* = 2–3; the data are representative of 2 independent experiments). The data are presented as the means. **C, D** Comparison of secretion levels between different cultures stimulated with **(C)** LPS or **(D)** TI (*n* = 3; the data are representative of 2 independent experiments). The data are presented as the means ± SDs. *p < 0.05, **p < 0.01, ***p < 0.001; one-way ANOVA with Tukey’s post hoc comparison. The dotted line indicates the maximum detection of the analyte. TI: TNF-α/IL-1β

We analyzed the inflammatory secretome with a multiplex assay to investigate the effects of different stimuli as well as glial interactions on inflammatory responses (Fig. 3A). LPS stimulation induced a strong inflammatory response in MG monocultures, resulting in increased secretion of chemokines (CXCL5, CCL2 and CXCL8) and cytokines (IL-6, IL-10, TNF-α, and IL-1β) compared with the control conditions (Fig. 3B, Supplementary Table 2). Similarly, TNF-α/IL-1β stimulation induced a strong inflammatory response in AS monocultures, resulting in prominent secretion of chemokines (CXCL10, CXCL5, CCL2, and CXCL8) and cytokines (GM-CSF and IL-6) as well as low secretion of IL-10 (Fig. 3B, Supplementary Table 2). Thus, microglia and astrocytes demonstrated cell type-specific responses to LPS and TNF-α/IL-1β stimulation, respectively. To confirm these findings, statistical tests were conducted between the stimulated MG and AS monocultures (Fig. 3C and D). Increased levels of secreted inflammatory factors were detected after LPS stimulation in MG monocultures (Fig. 3C) and after TNF-α/IL-1β stimulation in AS monocultures (Fig. 3D). IFN-γ stimulation, in turn, generated more moderate responses in both cell types, inducing the secretion of CXCL10, CCL2, CXCL8, and IL-1β in MG monocultures and minor changes in the secretion of CCL2, IL-6 and IL-10 in AS monocultures (Fig. 3B, Supplementary Fig. 6A, Supplementary Table 2).

Next, we wanted to understand how the inflammatory responses of microglia and astrocytes are modulated in cocultures. MG/AS cocultures exhibited patterns of cell type-specific inflammatory activation similar to those observed in monocultures (Fig. 3B, Supplementary Table 2). However, the secreted levels of several chemokines and cytokines were significantly altered, indicating reciprocal modulation of inflammatory responses between the cell types. Compared with MG monocultures, LPS stimulation of MG/AS cocultures induced greater secretion of CXCL10 and GM-CSF while resulting in lower secretion of several factors, including CCL2, IL-6, IL-10, TNF-α, and IL-1β (Fig. 3C). Interestingly, elevated levels of CXCL10 and GM-CSF were detected in TNF-α/IL-1β-stimulated AS monocultures but not in LPS-stimulated MG monocultures (Fig. 3B). These findings suggest that the increase of CXCL-10 and GM-CSF in LPS-stimulated MG/AS cocultures indicates the inflammatory activation of astrocytes through interaction with LPS-activated microglia.

In contrast, TNF-α/IL-1β stimulation induced greater secretion of IL-10 and lower secretion of GM-CSF in MG/AS cocultures than in AS monocultures (Fig. 3D). Similarly, since IL-10 was secreted primarily by LPS-stimulated MG monocultures, its secretion in TNF-α/IL-1β-stimulated MG/AS cocultures suggests that inflammatory activation of microglia occurs through interaction with inflammatory astrocytes. Thus, while LPS and TNF-α/IL-1β induced strong cell type-specific inflammatory responses in monocultures, the secretion of various chemokines and cytokines was altered in cocultures, demonstrating that glial interactions modulate inflammatory responses.

### Microfluidic coculture platform facilitates the study of microglial migration and glial activation within distinct inflammatory microenvironments

**Fig. 4:**
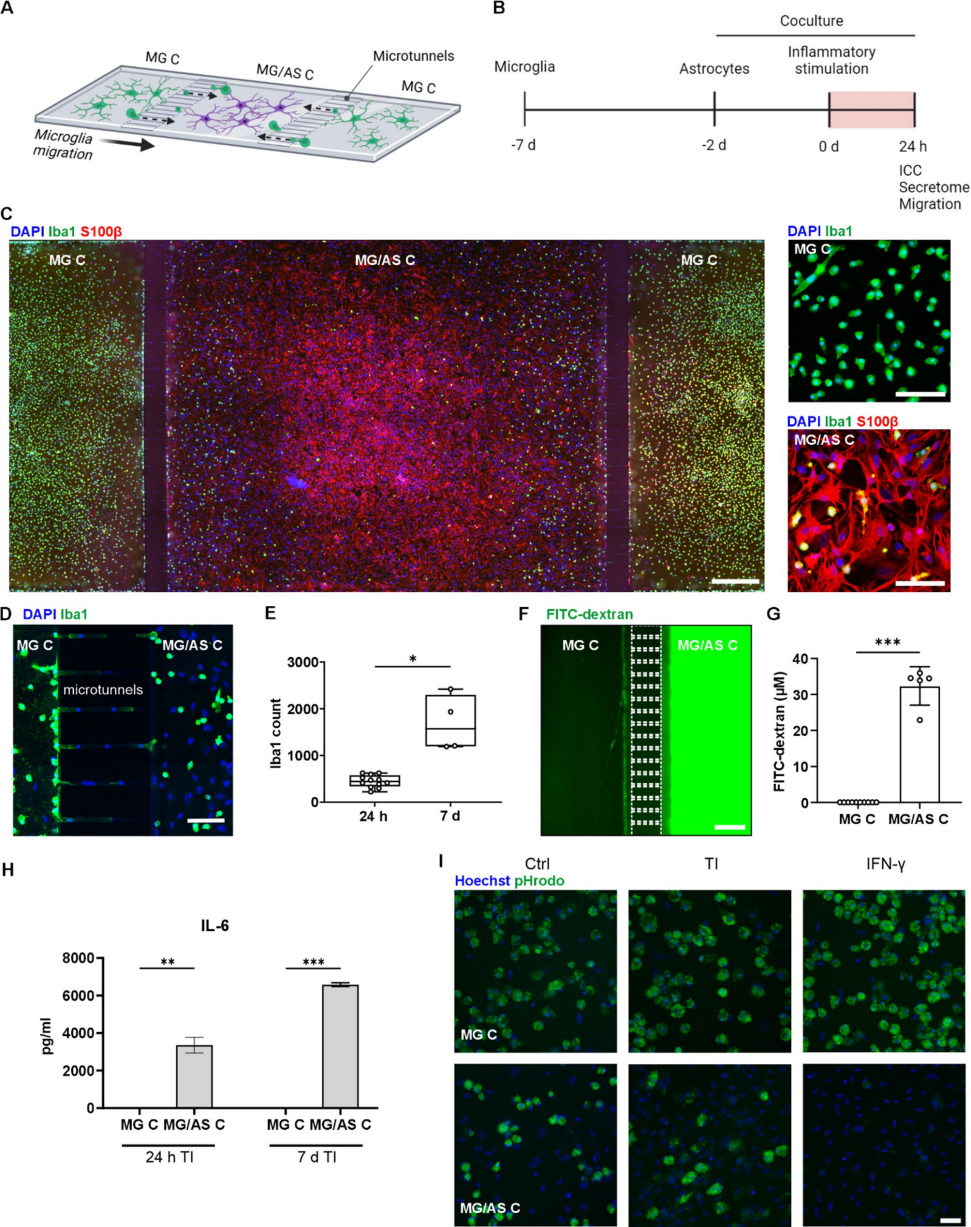
Establishment of a microfluidic coculture platform to study glial activation and microglial migration. **A** Schematic figure of the microfluidic coculture platform design. **B** Timeline of the experimental setup. Created in BioRender. Hagman, S. (2025) https://BioRender.com/loiww5m. **C** Representative immunofluorescence image of the cells cultured in the compartmentalized microfluidic platform. Scale bar is 500 μm. Close-up images of microglia in MG C and microglia and astrocyte cocultures in MG/AS C. Scale bar is 100 μm. **D** Representative immunofluorescence image showing close-up of Iba1-positive microglia migrating through the microtunnels. Scale bar is 100 μm. **E** Microglia demonstrated spontaneous migration toward the MG/AS C, which increased over time (n(24 h) = 12 with 6 platforms, n(7 d) = 4 with 2 platforms; migration through microtunnels analyzed separately for both sides). The data are presented as boxplots showing independent values and whiskers indicating min and max. **F** Representative fluorescence image showing no detectable diffusion of FITC-conjugated dextran from MG/AS C to MG C in the microfluidic platform after 24 h. Scale bar is 250 μm. **G** Measurement of the concentration of FITC-conjugated dextran from different compartments after 24 h (*n* = 5–10). The data are presented as the means ± SDs with individual values. **H** IL-6 secretion measured with ELISA in different compartments after 24 h and 7 d of selective MG/AS C inflammatory stimulation with TNF-α/IL-1β (TI) (*n* = 2). The data are presented as the means ± SDs. **I** Representative fluorescence images showing microglial phagocytosis of fluorescent pHrodo bioparticles in different compartments after 6 d of selective MG/AS C inflammatory stimulation with TNF-α/IL-1β (TI) or IFN-γ. Scale bar is 50 μm. *p < 0.05, **p < 0.01, ***p < 0.001; Student’s t test. MG C: microglia compartment; MG/AS C: microglia–astrocyte compartment

To better model glial interactions and the effects of inflammatory microenvironments on glial functions, we established a microfluidic coculture platform (Fig. 4A and B, Supplementary Fig. 1). The coculture platform consisted of three sequential cell compartments interconnected by microtunnels [30, 42]. This enabled the culture of microglia in their separate dedicated compartments (MG C), while the microtunnels facilitated microglial migration toward the microglia–astrocyte compartment (MG/AS C) (Fig. 4C and D, Supplementary Video 1), resulting in spontaneous formation of cocultures. The migration of microglia toward MG/AS C increased over an extended culture period (Fig. 4E). Each compartment was provided with its own media chamber to allow the culture of microglia and astrocytes in cell type-specific media as well as the establishment of separate inflammatory microenvironments to study inflammatory interaction. The fluidic isolation between the different compartments was tested using FITC-conjugated dextran of a size comparable to that of secreted cytokines (15–25 kDa) (Fig. 4F and G). The results revealed near-complete fluidic isolation with no detectable diffusion of fluorescent dextran between the compartments over a 24-hour test period.

To further confirm the establishment of separate microenvironments within the coculture platform, we stimulated only MG/AS C with TNF-α/IL-1β for 24 h and 7 d and measured the secretion of IL-6, a key cytokine highly upregulated in astrocytes after TNF-α/IL-1β stimulation, from both MG C and MG/AS C. Elevated IL-6 was detected only within the stimulated compartment, MG/AS C, ensuring that it did not diffuse at detectable levels into the neighboring compartment, MG C (Fig. 4H). In addition, the functionality of migrating microglia was evaluated via a pHrodo phagocytosis assay. We confirmed that microglia migrating to the MG/AS C performed phagocytic functions in the basal state (Fig. 4I). However, after targeted proinflammatory stimulation of MG/AS C with IFN-γ, we observed compromised phagocytic function in the inflammatory microenvironment. Thus, the microfluidic coculture platform demonstrated successful coculturing of microglia and astrocytes in nearly completely fluidically isolated compartments with intercompartmental microglial migration, allowing investigations of glial activation within distinct microenvironments.

### The chemoattractant ADP increases microglial migration in the microfluidic platform

**Fig. 5:**
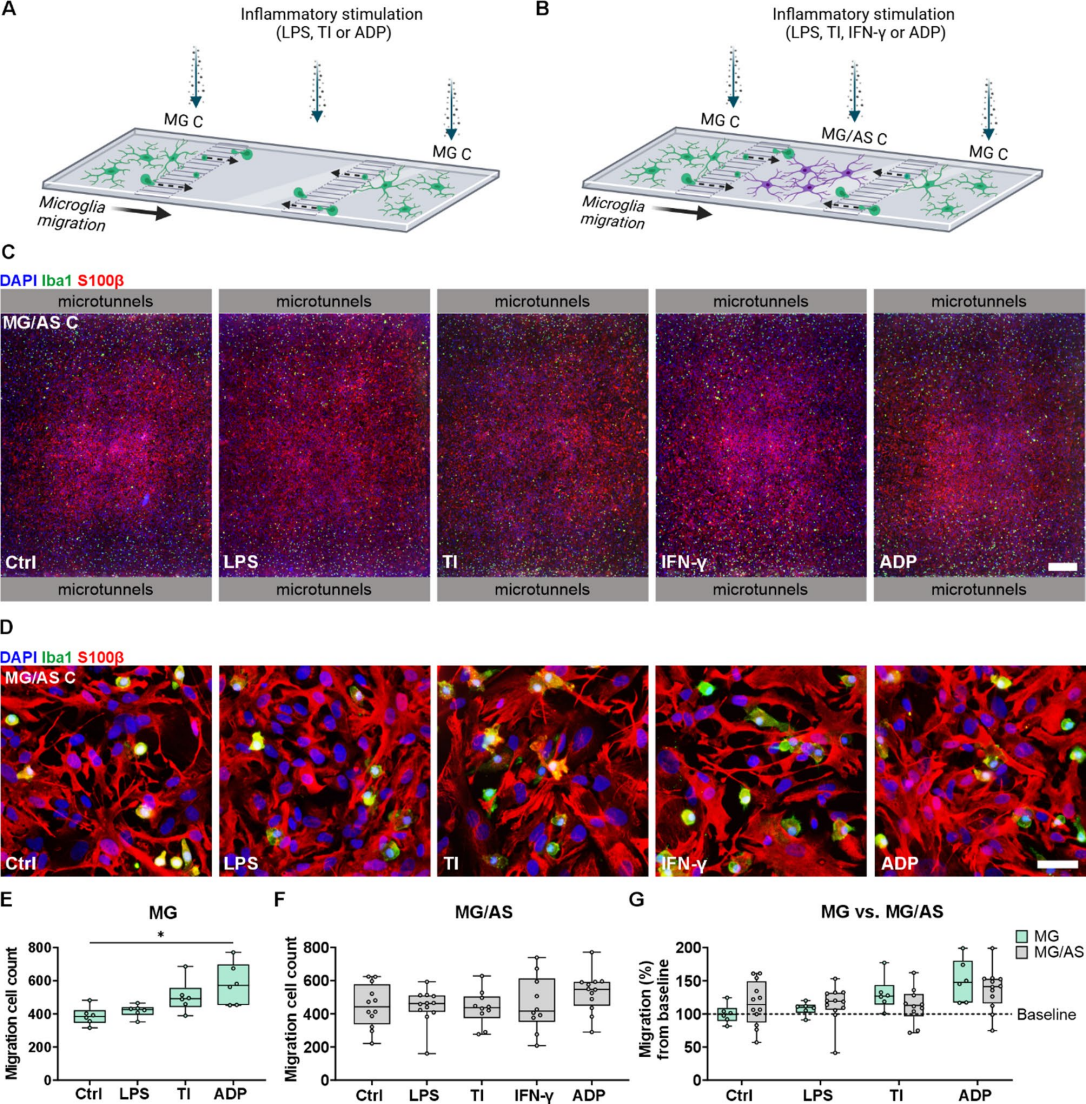
ADP increases microglial migration in the microfluidic platform. **A, B** Schematic figures of microglial migration in the microfluidic platform **(A)** without astrocytes (MG) and **(B)** with astrocytes (MG/AS). Created in BioRender. Hagman, S. (2025) https://BioRender.com/loiww5m. **C** Representative immunofluorescence images of the MG/AS C in the coculture platform after 24 h of inflammatory stimulation. Scale bar is 500 μm. **D** Representative immunofluorescence images showing close-up of cocultures in the MG/AS C after 24 h of inflammatory stimulation. Scale bar is 50 μm. **E** Cell count of microglia that migrated into the middle compartment in MG platforms **(A)** after 24 h of stimulation (*n* = 6 with 3 platforms in each group). Microglial migration increased significantly following ADP stimulation. *p < 0.05, Mann–Whitney U test. **F** Cell count of microglia that migrated into the MG/AS C in MG/AS platforms **(B)** after 24 h of stimulation (*n* = 10–12 with 5–6 platforms in each group, the data are representative of 2 independent experiments). No significant alterations in migration were detected between the control and stimulated groups. **G** Comparison of microglial migration in the MG **(A, E)** and MG/AS platforms **(B, F)** as a percentage of the MG baseline. Migration through the microtunnels was analyzed separately for both sides of the microfluidic platform. The data are presented as boxplots showing independent values and whiskers indicating min and max. MG C: microglia compartment; MG/AS C: microglia–astrocyte compartment; TI: TNF-α/IL-1β

To explore how inflammatory stimulation affects microglial migration in the microfluidic coculture platform, we investigated the number of Iba1-positive cells in the middle MG/AS C after 24 h of stimulation of all compartments (Fig. 5A–D). In addition to different inflammatory stimuli, ADP, a known activator of microglial migration [45], was also used as a positive control. First, we studied the effects of the stimuli on microglia cultured in the microfluidic platform without astrocytes (Fig. 5A). Microglia increased their migration from MG C toward the middle compartment after 24 h of ADP stimulation of all compartments (*p* < 0.05) (Fig. 5E). There was also a modest increase in migration in response to TNF-α/IL-1β, but this increase was not statistically significant. In contrast, in the presence of astrocytes (Fig. 5B) microglial migration toward MG/AS C was more variable than that of microglia cultured alone. Although no statistically significant differences were detected between the stimulation groups, a trend of increased migration following the addition of ADP was noted (Fig. 5F and G). Thus, while the presence of astrocytes and proinflammatory challenge did not result in major changes in microglial migration, we demonstrated an increase in microglial migration upon exposure to the ADP stimulus.

### Cell type-specific inflammatory responses and glial interactions are demonstrated in the microfluidic coculture platform

**Fig. 6:**
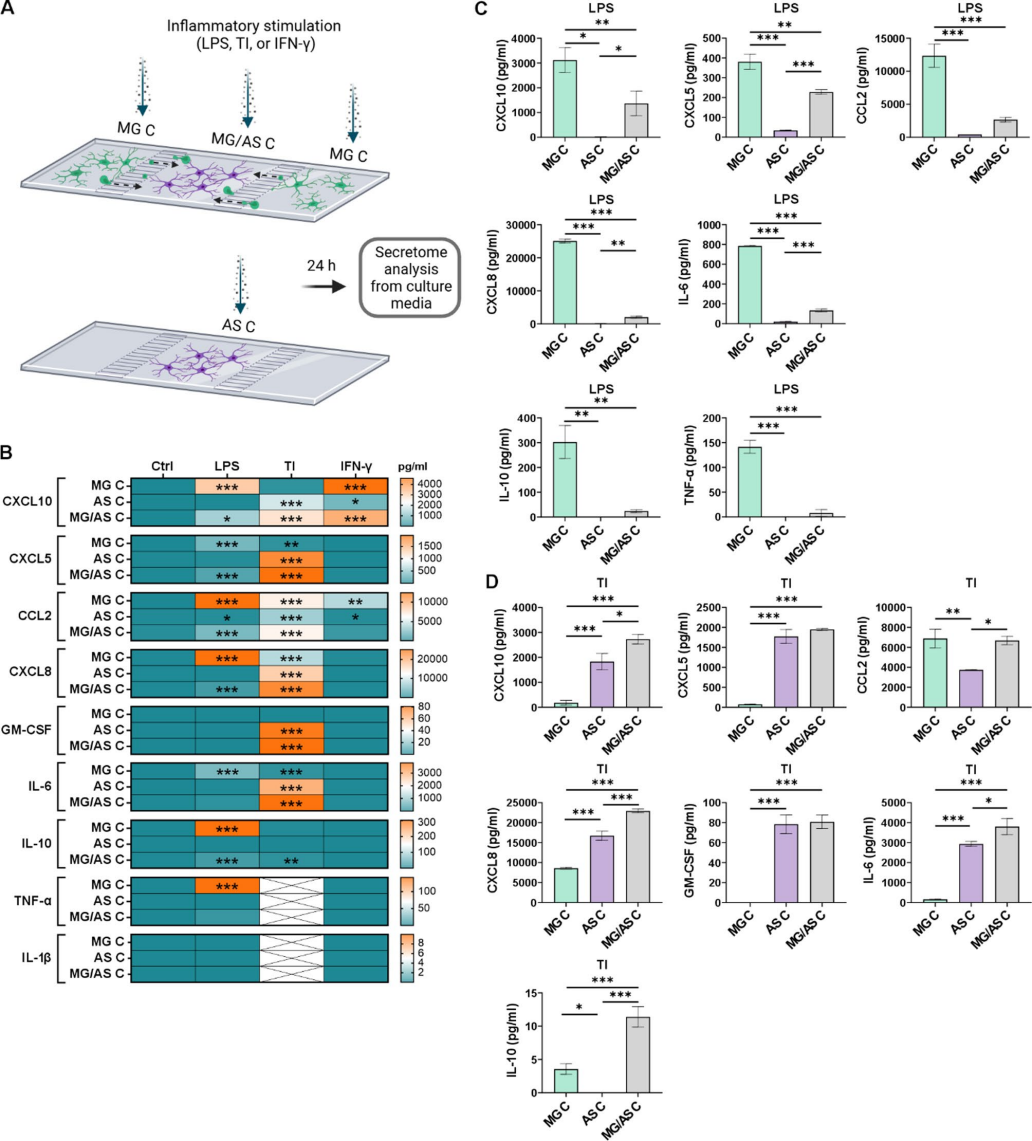
Inflammatory stimulation of the microfluidic coculture platform replicates cell type-specific responses and glial crosstalk. **A** Schematic figure of the coculture setup and inflammatory stimulation. Created in BioRender. Hagman, S. (2025) https://BioRender.com/loiww5m. **B** Heatmap of the secretion of inflammatory cytokines and chemokines in the microfluidic platform after 24 h of stimulation. Asterisks indicate statistical significance in comparison with the control. **C, D** Comparison of secretion levels between different compartments stimulated with **(C)** LPS or **(D)** TI. Secretion of GM-CSF and IL-1β was not detected in the compartments after LPS stimulation. The dotted line indicates the maximum detection of the analyte (*n* = 2–3; the data are representative of 2 independent experiments except for AS C, which was performed once). The data are presented as the means ± SDs. *p < 0.05, **p < 0.01, ***p < 0.001; one-way ANOVA with Tukey’s post hoc comparison. AS C: astrocyte compartment; MG C: microglia compartment; MG/AS C: microglia–astrocyte compartment; TI: TNF-α/IL-1β

To verify that we can reproduce the observed inflammatory activation and interactions of microglia and astrocytes in the microenvironments of the microfluidic coculture platform, we stimulated both MG Cs and MG/AS C and analyzed the secretion of inflammatory mediators (Fig. 6A). To mimic the conventional culture setup and associate the results of conventional cultures with cultures in the microfluidic platform, we also included microfluidic platforms that contained only astrocytes in their designated compartment (AS C). As in conventional monocultures (Fig. 3B), both LPS and TNF-α/IL-1β stimulation induced strong cell type-specific immune responses in the corresponding compartments, MG C and AS C (Fig. 6B, Supplementary Table 3). Accordingly, IFN-γ stimulation induced minor responses in MG C and AS C (Fig. 6B, Supplementary Fig. 6B).

Compared with conventional cultures, the cultures in the microfluidic platform presented considerably lower levels of secretion, reflecting overall smaller cell quantities (Figs. 3B–D and 6B–D, Supplementary Tables 2 and 3). Therefore, statistical tests were not performed between the conventional cultures and the microfluidic coculture platform. However, the calculated fold change (FC) values provide an indication of the magnitude of changes occurring in both culture setups (Supplementary Table 4). Cocultures of MG/AS C produced similar patterns of LPS- and TNF-α/IL-1β -induced responses as conventional MG/AS cocultures, confirming the contribution of migrated microglia to inflammatory activation (Figs. 3B–D and 6B–D, Supplementary Table 4). Furthermore, inflammatory interaction between glial cells was evidenced by increased IL-10 secretion in MG/AS C compared with AS C (FC 11.5) following TNF-α/IL-1β stimulation (Fig. 6D). This pattern was similar to that observed in conventional MG/AS cocultures compared with AS monocultures (FC 74.7) (Fig. 3D, Supplementary Tables 2–4). Overall, these results demonstrate that microglia and astrocytes cocultured in the microfluidic platform reproduce the inflammatory activation patterns observed in conventional monocultures and cocultures and can reveal glial interaction.

### Glial interactions elevate complement component C3 levels in cocultures

**Fig. 7:**
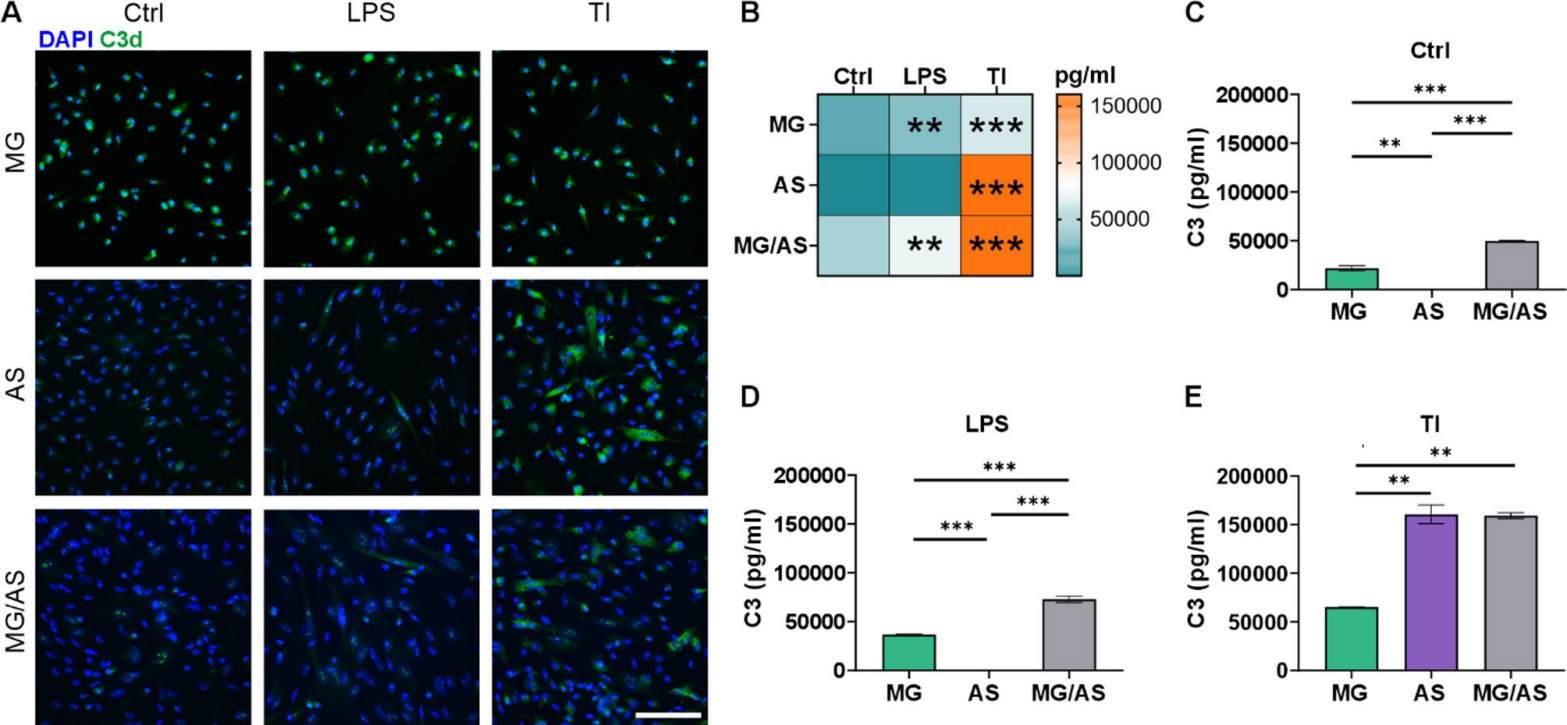
Enhanced complement component 3 (C3) secretion driven by glial interaction in cocultures. **A** Representative images of immunocytochemical staining for C3d in MG and AS monocultures and MG/AS cocultures after 24 h of inflammatory stimulation. Scale bar is 100 μm. **B** Heatmap of C3 secretion in MG and AS monocultures and MG/AS cocultures after 24 h of stimulation. The data are presented as the means. Asterisks indicate statistical significance in comparison with the control condition. **C–E** Secretion of C3 in MG and AS monocultures and MG/AS cocultures (**C**) under control conditions and after stimulation with (**D**) LPS or (**E**) TI. The data are presented as the means ± SDs (*n* = 2). *p < 0.05, **p < 0.01, ***p < 0.001; one-way ANOVA with Tukey’s post hoc comparison. TI: TNF-α/IL-1β

Since reciprocal modulation of inflammatory responses was observed in glial cocultures, we wanted to further investigate the inflammatory interactions between microglia and astrocytes by analyzing complement C3 expression in the stimulated cultures. Complement system activation has been highlighted as one mechanism by which microglia and astrocytes communicate and potentially participate in the progression of neuroinflammatory pathologies [15, 24]. Immunocytochemical staining of C3d, a downstream product of C3, revealed high expression in MG monocultures at the basal level and after LPS and TNF-α/IL-1β stimulation (Fig. 7A). AS monocultures and MG/AS cocultures, respectively, demonstrated modest/non-detectable expression of C3d under control conditions. While LPS stimulation had no observable effects, TNF-α/IL-1β stimulation demonstrated strong responses in AS monocultures and MG/AS cocultures, suggesting high expression of C3 in inflammatory astrocytes.

Analysis of C3 secretion levels in the cultures provided further support for the observed staining results (Fig. 7B–E). In the basal state, only MG monocultures and MG/AS cocultures secreted C3, and the secretion increased with both LPS and TNF-α/IL-1β stimulation (Fig. 7B). Interestingly, a greater level of C3 was observed in MG/AS cocultures than in MG monocultures both under control conditions and after LPS stimulation (Fig. 7C and D), indicating the amplification of C3 through microglial crosstalk with astrocytes. As observed with C3 staining, AS monocultures and MG/AS cocultures presented highly elevated secretion of C3 in response to TNF-α/IL-1β stimulation (Fig. 7B and E). Overall, both MG and AS monocultures, as well as MG/AS cocultures, demonstrated the highest levels of C3 secretion with TNF-α/IL-1β stimulation (Fig. 7B–E), suggesting that TNF-α and/or IL-1β are potent activators of the complement system in both glial cell types. In summary, these results demonstrated complement activation in our glial cultures after inflammatory stimulation as well as modulation of C3 expression via glial interaction.

## Discussion

Increasing evidence indicates a central role of microglia- and astrocyte-driven neuroinflammatory processes in the progression of various neurodegenerative pathologies [2, 46, 47]. However, an insufficient understanding of how microglia and astrocytes communicate and mediate inflammatory responses highlights the necessity of developing in vitro models that are more physiologically relevant to humans. To address these issues, we established in vitro glial coculture models to investigate the function and interactions of human iPSC-derived microglia and astrocytes under inflammatory conditions. We cocultured glial cells in both conventional culture dishes and a microfluidic coculture platform and stimulated the cells with LPS, TNF-α/IL-1β or IFN-γ. LPS is a widely used stimulus of microglial activation and triggers the production and secretion of proinflammatory factors such as TNF-α and IL-1β [6, 48, 49], while TNF-α and IL-1β play significant roles in the activation of astrocytes [8, 50]. IFN-γ, in turn, mediates glial activation during neuroinflammatory conditions, including MS [51, 52]. Here, microglia and astrocytes demonstrated strong cell type-specific responses to LPS and TNF-α/IL-1β stimulation that were modulated under coculture conditions, indicating reciprocal signaling between glial cells. In contrast to conventional cultures, the microfluidic coculture platform provides a more functional approach for investigating glial interactions, enabling the establishment of separate inflammatory microenvironments with spontaneous microglial migration.

The inflammatory responses of microglia and astrocytes have been studied extensively in monocultures via the use of various inflammatory stimuli [7, 8, 15, 17, 49, 53]. However, accumulating evidence from coculture studies indicates the importance of cell–cell contacts in revealing physiologically relevant inflammatory functions of glial cells [9, 23, 24, 54]. To understand how the interplay between microglia and astrocytes modulates the inflammatory responses, we first measured the secretion of inflammatory mediators from the culture media of both conventional microglia and astrocyte monocultures and glial cocultures. In line with previous studies [6, 37, 49, 50], microglia and astrocyte monocultures responded to LPS and TNF-α/IL-1β stimulation, respectively, via the secretion of a wide range of chemokines and cytokines. The observed responses demonstrated cell type specificity, with only minor effects of TNF-α/IL-1β stimulation on microglia and LPS stimulation on astrocytes. Accordingly, LPS induced activation of microglia is known to occur via pattern recognition receptors, such as Toll-like receptor 4 (TLR4) [55], while human astrocytes have been reported to differ in their expression of TLR4 from their mouse counterparts [56, 57] and do not respond to LPS stimulus [10, 56]. Therefore, it should be acknowledged that inflammatory responses may be influenced by species-specific differences but also gender-specific variations.

While both LPS and TNF-α/IL-1β stimuli elicited broad responses in glial cocultures, alterations in secretion levels suggested the modulation of inflammatory responses via reciprocal signaling. Compared with MG monocultures, LPS stimulation of cocultures resulted in reduced secretion of various inflammatory mediators, indicating that microglial inflammatory responses are dampened when cocultured with astrocytes. This could indicate interplay between glial cells via the anti-inflammatory cytokine IL-10 secreted by LPS-stimulated microglia. IL-10 has been reported to evoke anti-inflammatory signaling between microglia and astrocytes to reduce inflammatory activation [58]. An increase in IL-10 secretion was also observed in TNF-α/IL-1β-stimulated cocultures compared with AS monocultures, which was indicative of microglial activation through inflammatory astrocytes. Conversely, LPS stimulation induced the secretion of CXCL10 and GM-CSF in cocultures, which was observed predominantly in TNF-α/IL-1β-stimulated AS monocultures, indicating the activation of astrocytes through inflammatory microglia. Previous studies have reported that reactive astrocytes secrete CXCL10 and GM-CSF in chronic CNS neuroinflammation, potentially enhancing the activation of astrocytes as well as the activation of microglia [59–61]. Therefore, these results confirm the intricate reciprocal modulation of inflammatory responses between cocultured microglia and astrocytes.

Since conventional cultures cannot recapitulate the complexity of glial functions within the CNS environment, we next cocultured microglia and astrocytes in a compartmentalized microfluidic coculture platform. This approach provided increased control over the conventional culture setup as microglia and astrocytes were cocultured in dedicated compartments and cell type-specific culture media. The microfluidic platform design facilitated investigation of glial interaction at a more functional level as migration of microglia through microtunnels toward astrocytes enabled the spontaneous formation of glial cocultures. Furthermore, near-complete fluidic isolation in the platform achieved through the narrow and shallow microtunnels [62, 63], supported the establishment of separate inflammatory microenvironments. This was demonstrated by the selective stimulation of the middle compartment containing both microglia and astrocytes with TNF-α/IL-1β. The stimulation induced high secretion of IL-6, which did not diffuse at detectable levels to the side compartments over a 7-d extended stimulation period. We further demonstrated the functional potential of the coculture platform with selective IFN-γ stimulation, which has previously been reported to suppress microglial phagocytosis [17, 21, 64]. This stimulation markedly reduced phagocytic function of migrated microglia without affecting microglia in unstimulated side compartments. When treated with LPS, TI, or IFN-γ stimuli, many of the reported astrocyte and microglia monoculture responses from conventional cultures were reproduced within the microfluidic platform. Additionally, reciprocal signaling was evidenced by IL-10 potentiation in TNF-α/IL-1β-stimulated cocultures, confirming the applicability of the platform for investigating glial interactions. We were unable to replicate the glial interactions in the microfluidic platform following LPS stimulation, as indicated by the increased CXCL10 and GM-CSF levels seen in conventional cultures. This may be due to the significantly higher number of microglia in the side compartment (3000 cells) compared to the middle compartment (900–1000 cells) of the microfluidic platform, where cocultures form spontaneously as a result of microglial migration. Given that LPS is a microglia-specific stimulus, measuring functional effects in astrocytes following interaction with microglia would require a higher density of microglia in the coculture.

As highly motile cells, microglia respond to neuroinflammatory events by migrating toward sites of inflammatory insult and pathological stimuli [46]. Therefore, we investigated how the inflammatory stimuli affected microglial migration rates in our microfluidic coculture platform. Previous studies have demonstrated conflicting effects of LPS stimulus on microglial migration, with some reporting increased [65, 66] and others decreased chemokinesis and chemotaxis [67–69]. Combined stimulation with TNF-α and IFN-γ, in turn, has been reported to decrease microglial migration [69, 70]. However, these studies were conducted utilizing rodent microglia and simple scratch-wound or transwell migration assays involving only one cell type. Here, we showed that the inflammatory stimuli LPS and IFN-γ had no significant effects on the microglial migration rate. Interestingly, when microglial migration was studied in the absence of astrocytes, a modest, although statistically insignificant, increase in migration in response to TNF-α/IL-1β was observed. The presence of astrocytes seemed to increase the variability in microglial migration, which may have affected the significance of the results. One well-recognized stimulus of microglial migration is extracellular nucleotide adenosine triphosphate (ATP), which participates in microglial recruitment toward CNS injuries [71–74]. Since ATP and the downstream product ADP have been shown to increase both the chemokinesis and chemotaxis of microglia in vitro [6, 17, 45, 75], we utilized ADP stimulation as a positive control in our microfluidic coculture platform. Accordingly, our results demonstrated that the migration of microglia increased after ADP stimulation, confirming the potential of our coculture platform for migration studies.

The complement cascade is a key regulator of innate immune responses [76]. Activation of the complement system mediates crosstalk between microglia and astrocytes during neuroinflammatory responses [15, 24] and has also been demonstrated in several neuroinflammatory diseases, including AD [77] and MS [78]. In this study, we showed that microglia, but not astrocytes, expressed C3 in the basal state. However, following inflammatory activation, astrocytes expressed C3 more prominently than microglia. Consistent with our results, previous publications have shown that elevated C3 is characteristic of reactive neuroinflammatory astrocytes in vitro and in vivo [15, 50]. Here, coculturing microglia and astrocytes elicited a significant increase in C3 secretion in comparison with monocultures in both the basal state and after LPS stimulation, demonstrating aggravation via reciprocal signaling between microglia and astrocytes. Accordingly, C3 secretion was recently reported to be mediated via glial crosstalk in neuronal tricultures by Guttikonda and colleagues [24]. Our results therefore support the role of C3 in inflammatory glial interaction, as C3 was upregulated in both microglia and astrocytes upon inflammatory activation and further potentiated in glial cocultures.

## Conclusions

Taken together, our iPSC-based coculture models confirm the inflammatory molecular conversation between microglia and astrocytes, revealing cell type-specific inflammatory responses as well as reciprocal signaling that altered the levels of secreted chemokines and cytokines in glial cocultures. Furthermore, by coculturing microglia and astrocytes in a compartmentalized microfluidic coculture platform, we were able to explore inflammatory glial interactions at a more functional level within distinct, interconnected inflammatory microenvironments. Here, microglia efficiently migrated toward astrocytes, forming spontaneous glial cocultures. Targeted inflammatory stimulation of a single compartment validated the creation of separate inflammatory environments within the coculture platform, as evidenced by distinct phagocytosis and secretion patterns in the neighboring compartments. Moreover, the microfluidic coculture platform successfully demonstrated its applicability for migration studies in response to the chemoattractant ADP and reaffirmed the inflammatory activation and interaction between microglia and astrocytes. Our results further demonstrated elevated secretion of C3 within glial cocultures upon inflammatory activation, emphasizing the role of C3 in inflammatory glial crosstalk. Our in vitro microfluidic coculture platform emerges as a novel tool for unraveling the mechanisms of glial function and interplay in neuroinflammation.

## Electronic supplementary material

Below is the link to the electronic supplementary material.

Supplementary Material 1

Supplementary Material 2

Supplementary Material 3

## Data Availability

No datasets were generated or analysed during the current study.

## Abbreviations

AD: Alzheimer’s disease
ADP: Adenosine diphosphate
AM: Astrocyte medium
AS: Astrocyte
AS C: Astrocyte compartment
ATP: Adenosine triphosphate
CNS: Central nervous system
C3: Complement component 3
EMP: Erythromyeloid progenitor cell
IPSC: Induced pluripotent stem cell
IFN: Interferon
IL: Interleukin
LPS: Lipopolysaccharide
MG: Microglia
MG C: Microglia compartment
MG/AS: Microglia/astrocyte
MG/AS C: Microglia–astrocyte compartment
MS: Multiple sclerosis
NES cell: Neuroepithelial stem cell
PD: Parkinson’s disease
PDMS: Polydimethylsiloxane
PM: Primitive macrophage
PVP: Polyvinylpyrrolidone
TI: TNF-α/IL-1β
TLR4: Toll-like receptor 4
TNF: Tumor necrosis factor
