# Supplementary material for: Modeling neuroinflammatory interactions between microglia and astrocytes in a human iPSC-based coculture platform
Source: Cell Commun Signal. 2025 Jun 20;23:298. doi: 10.1186/s12964-025-02304-x (PMC12181861; doi:10.1186/s12964-025-02304-x)
Supplement: Supplementary file 2 — Supplementary Material 2 [file 12964_2025_2304_MOESM2_ESM.docx]

Supplementary Video 1. Time-lapse of microglial migration through the microtunnels in the microfluidic platform. The images were obtained during 1 hour with a 10-minute interval. The yellow arrow indicates a microglia migrating through the microtunnel. The green and blue arrows indicate microglia sensing the microtunnel entrance.
