# Supplementary material for: Modeling neuroinflammatory interactions between microglia and astrocytes in a human iPSC-based coculture platform
Source: Cell Commun Signal. 2025 Jun 20;23:298. doi: 10.1186/s12964-025-02304-x (PMC12181861; doi:10.1186/s12964-025-02304-x)
Supplement: Supplementary file 3 — Supplementary Material 3 [file 12964_2025_2304_MOESM3_ESM.docx]

**Supplementary material**

**Modeling neuroinflammatory interactions between microglia and astrocytes in a human iPSC-based coculture platform**

Iisa Tujula^1^*, Tanja Hyvärinen^1^*, Johanna Lotila^1^, Julia Rogal^2,3,4,^ Dimitrios Voulgaris^2,3,4^, Lassi Sukki^5^, Kaisa Tornberg^5^, Katri Korpela^1^, Henna Jäntti^6^, Tarja Malm^6^, Anna Herland^2,3,4^, Pasi Kallio^5^, Susanna Narkilahti^7^, Sanna Hagman^1^

*Contributed equally to the study.

# **Supplementary Figures**


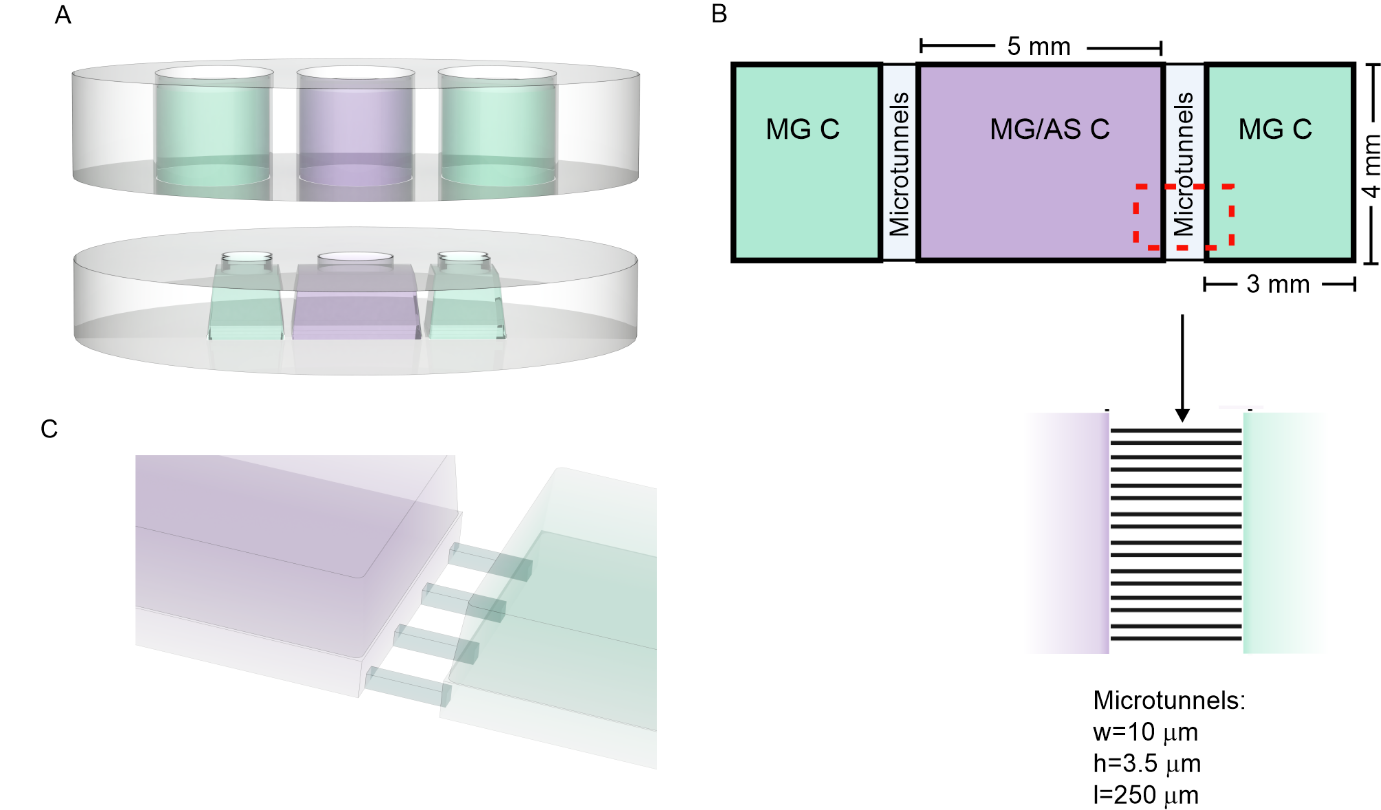


**Supplementary Fig. 1** Design of the microfluidic coculture platform**.** **A** The microfluidic platform is composed of two separate PDMS parts: a medium reservoir part (upper) and a cell culture part (lower) containing three cell compartments. **B** The microglia–astrocyte compartment (MG/AS C) is interconnected with 40 microtunnels to two microglia compartments (MG C). The dimensions of the cell compartments and microtunnels (l=length; h=height, w=width) are described in the image. **C** Close-up image showing microtunnels between the cell compartments.


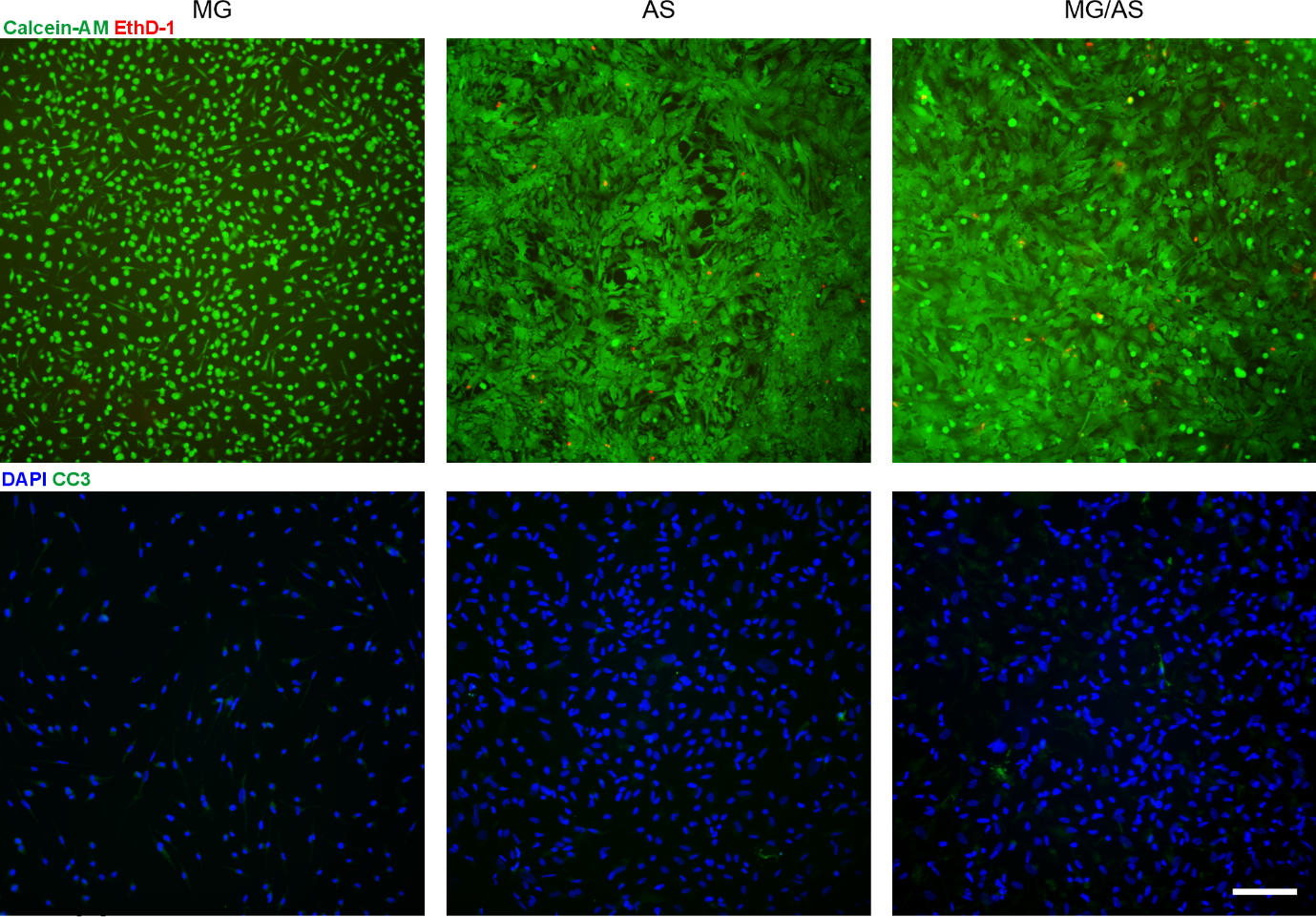


**Supplementary Fig. 2** Viability of glial monocultures and cocultures. Representative immunofluorescence images of cells stained with the viability markers calcein-AM and EthD-1 and the apoptotic marker cleaved caspase-3 (CC3). Scale bar is 100 µm.


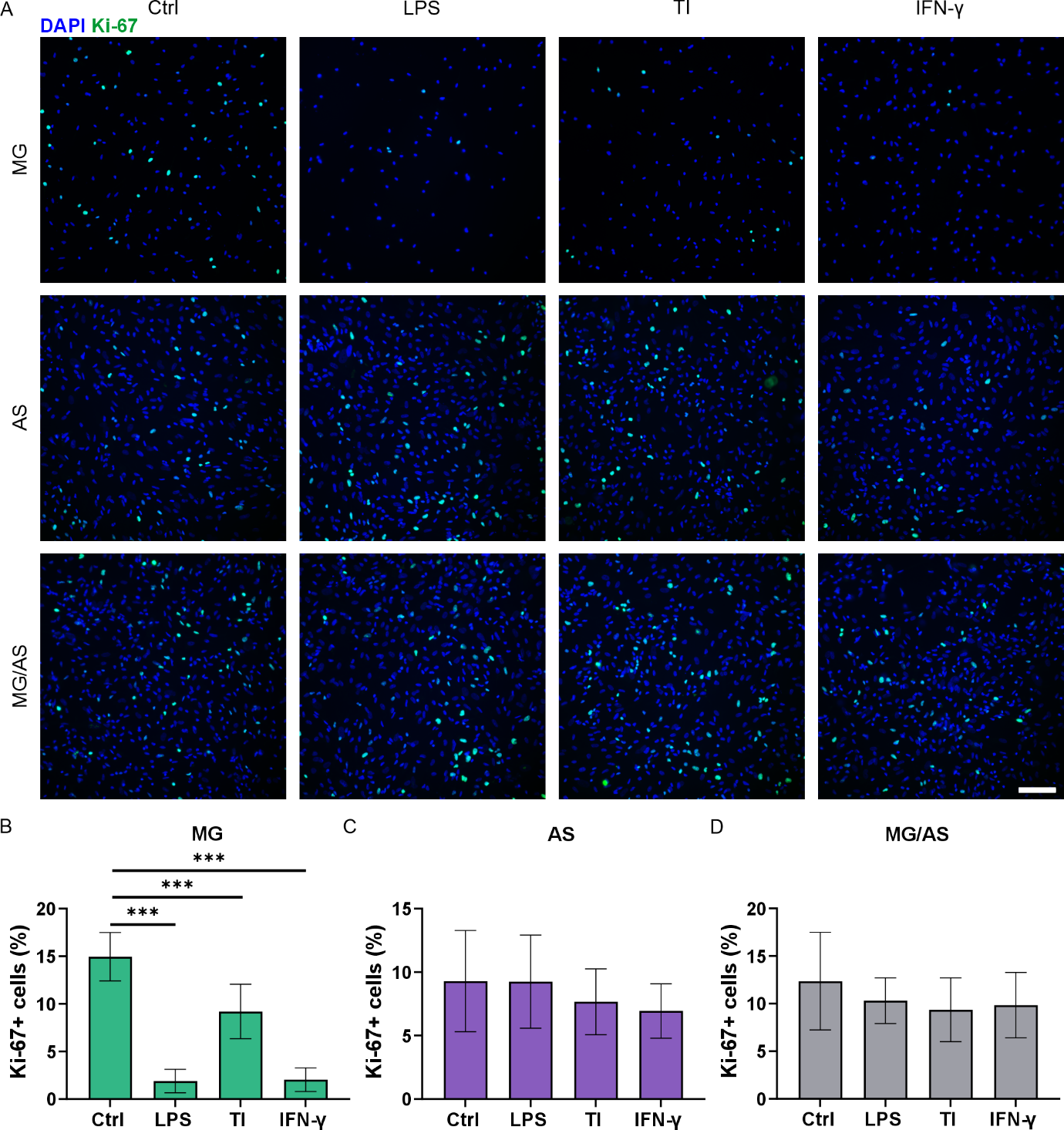


**Supplementary Fig. 3** Proliferation of glial cultures after 24 h of inflammatory stimulation. **A** Representative images of immunocytochemical staining for the proliferation marker Ki-67. Scale bar is 100 µm. **B–D** Ki-67-positive (+) cells were quantified from cultures as a percentage (%) of the total cell count. n = 18 images per condition; images from 2 independent experiments. The data are presented as the means ± SDs. ***p < 0.001; one-way ANOVA with Tukey’s post hoc comparison.


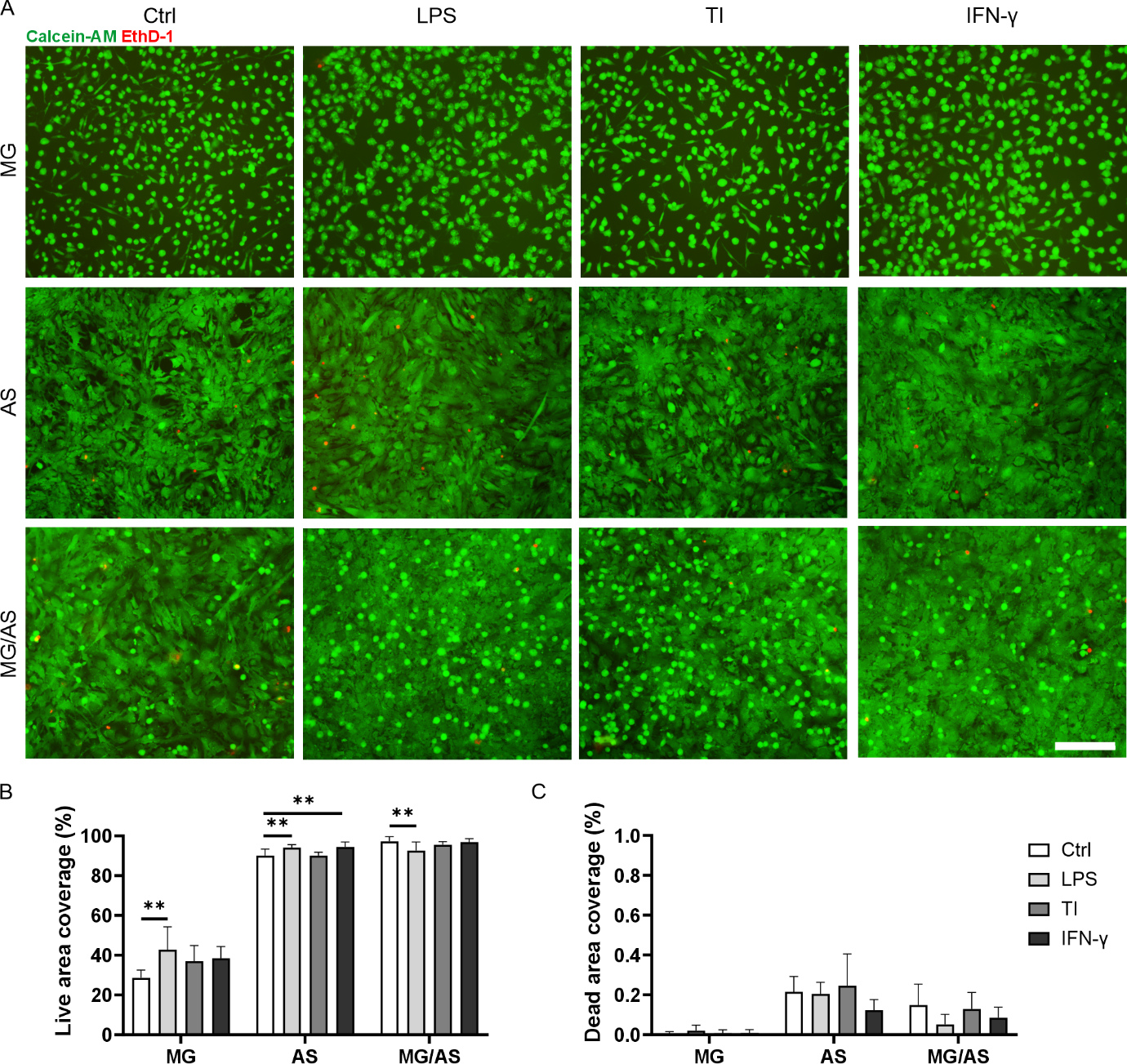


**Supplementary Fig. 4** Viability of glial cultures after 24 h of inflammatory stimulation. **A** Representative immunofluorescence images of cells stained with the viability markers calcein-AM and EthD-1. Scale bar is 200 µm. **B** Area covered by calcein-AM positive live cells as percentage (%) of the total area. **C** Area covered by EthD-1 positive dead cells as percentage (%) of the total area. n = 9 images per condition. The data are presented as the means ± SDs. **p < 0.01; one-way ANOVA with Tukey’s post hoc comparison.


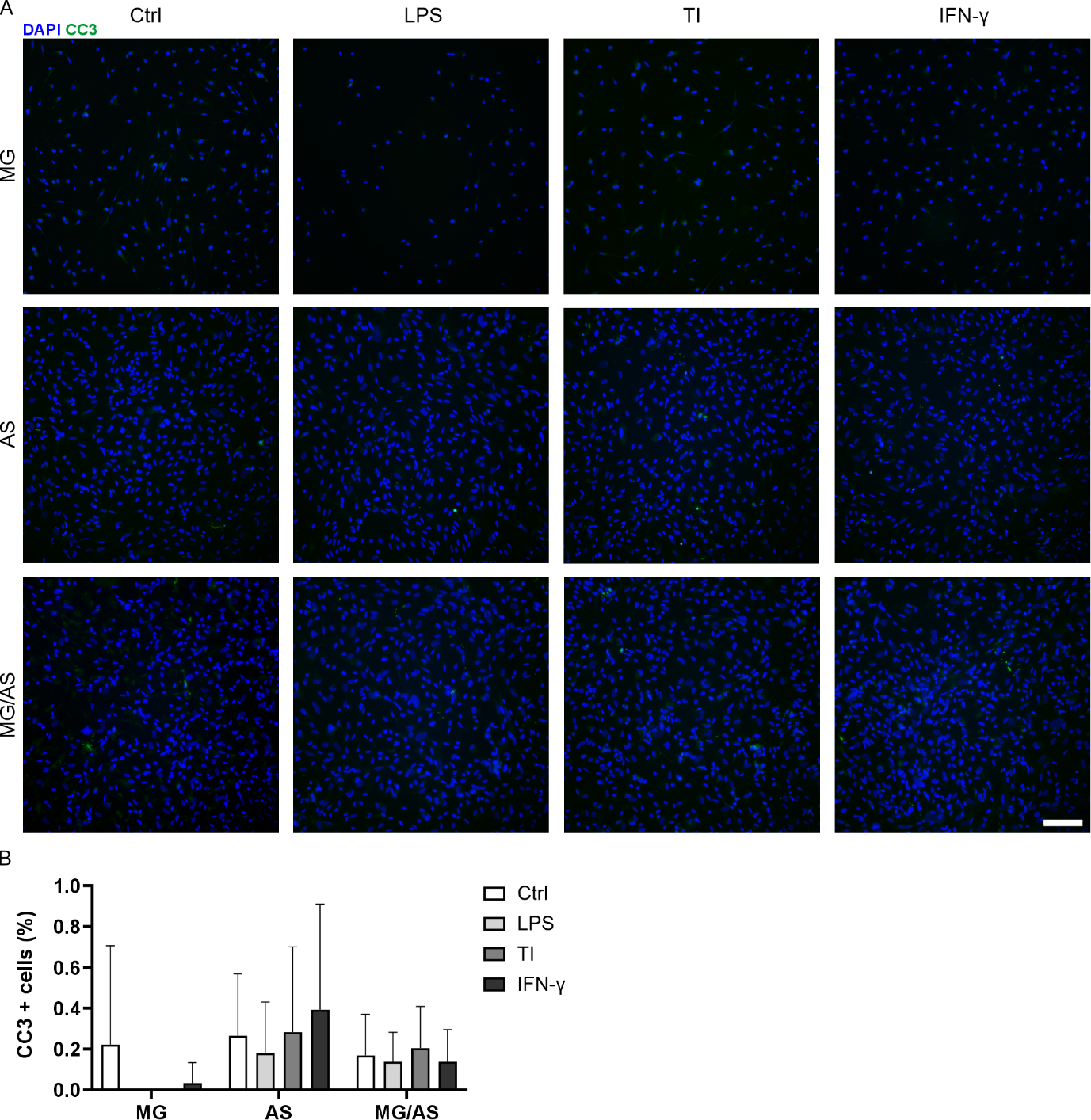


**Supplementary Fig. 5** Expression of cleaved caspase-3 (CC3) in glial cultures after 24 h of inflammatory stimulation**. A** Representative images of immunocytochemical staining for the apoptotic marker CC3. Scale bar is 100 µm. **B** CC3 positive (+) cells as percentage (%) of the total cell count. n = 9 images per condition. The data are presented as the means ± SDs. p > 0.05; one-way ANOVA with Tukey’s post hoc comparison.

**
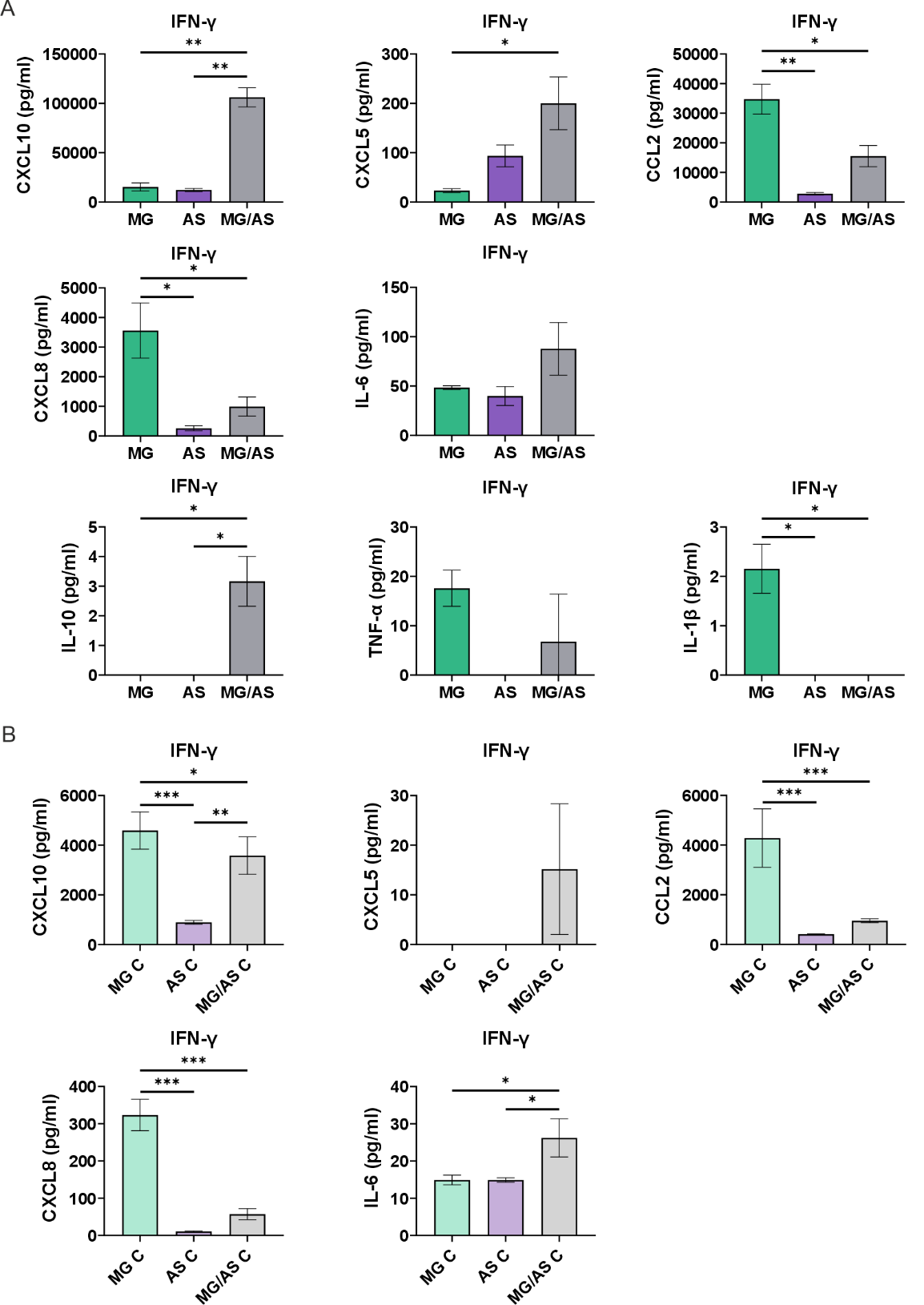
**

**Supplementary Fig. 6** Inflammatory secretion in glial cultures after 24 h of IFN-γ stimulation. **A** Comparison of the secretion levels of cytokines and chemokines between conventional monocultures and cocultures stimulated with IFN-γ (n = 2; the data are representative of 2 independent experiments). Secretion of GM-CSF was not detected in the cultures after IFN-γ stimulation. The data are presented as the means ± SDs. **B** Comparison of the secretion levels of cytokines and chemokines between different cell compartments in the microfluidic platform after IFN-γ stimulation (n = 2–3; the data are representative of 2 independent experiments). Secretion of GM-CSF, IL-10, TNF-α and IL-1β was not detected in the compartments after IFN-γ stimulation. The data are presented as the means ± SDs. *p < 0.05, **p < 0.01, ***p < 0.001; one-way ANOVA with Tukey’s post hoc comparison.

# **Supplementary Tables**

**Supplementary Table 1.** Primary and secondary antibodies used for the immunocytochemical stainings.

| **Primary antibody** | **Animal** | **Product number** | **Producer** | **Dilution (conventional cultures)** | **Dilution (microfluidic platform)** |
| --- | --- | --- | --- | --- | --- |
| Iba1 | Rabbit | 019-19741 | FujiFilm Wako | 1:500 | 1:200 |
| TMEM119 | Rabbit | ab185333 | Sigma-Aldrich | 1:100 | - |
| P2RY12 | Rabbit | HPA014518 | Sigma-Aldrich | 1:125 | - |
| CD44 | Rabbit | ab157107 | Abcam | 1:500 | - |
| S100β | Mouse | S2532 | Sigma-Aldrich | 1:500 | 1:200 |
| GFAP | Chicken | ab4674 | Abcam | 1:4000 | - |
| Ki-67 | Rabbit | AB9260 | Millipore | 1:800 | - |
| Cleaved Caspase-3 | Rabbit | 9664 | Cell signaling Technology | 1:400 | - |
| C3d complement | Rabbit | A0063 | Agilent Technologies | 1:2000 | - |
| **Secondary antibody** | **Animal** | **Product number** | **Producer** | **Dilution (conventional cultures)** | **Dilution (microfluidic platform)** |
| Alexa Fluor 488 donkey anti-rabbit IgG (H+L) | Donkey | A21206 | Thermo Fisher Scientific | 1:400 | 1:200 |
| Alexa Fluor 488 donkey anti-mouse IgG | Donkey | A21202 | Thermo Fisher Scientific | 1:400 | 1:200 |
| Alexa Fluor 568 donkey anti-mouse IgG | Donkey | A10037 | Thermo Fisher Scientific | 1:400 | 1:200 |
| Alexa Fluor 568 donkey anti-rabbit IgG | Donkey | A10042 | Thermo Fisher Scientific | 1:400 | 1:200 |
| Alexa fluor 647 goat anti-chicken IgY | Goat | A21449 | Thermo Fisher Scientific | 1:200 | - |

**Supplementary Table 2.** Inflammatory secretion (mean ± SD) in control and stimulated conventional astrocyte (AS) and microglia (MG) monocultures and cocultures (MG/AS) measured with a multiplex assay. The data are represented as the means of samples ± SDs (n= 2–3 with 2 technical replicates).

| **Analyte (pg/mL)** | **Culture** | **Ctrl** | **LPS** | **TNF-α/IL-1β** | **IFN-γ** |
| --- | --- | --- | --- | --- | --- |
| CXCL10 | MG | 33.4 ± 6.2 | 4086.9 ± 349.1 | 126.4 ± 17.0 | 15259.2 ± 4124.3 |
|  | AS | 30.3 ± 4.5 | 600.2 ± 124.3 | 79765.9 ± 13012.6 | 12254.8 ± 1582.7 |
|  | MG/AS | 159.8 ± 43.3 | 48396.7 ± 967.1 | 83268.2 ± 7618.6 | 106130.1 ± 9715.5 |
| CXCL5 | MG | 7.9 ± 6.9 | 3996.5 ± 527.5 | 572.9 ± 97.6 | 23.3 ± 3.8 |
|  | AS | 111.1 ± 11.9 | 417.3 ± 63.0 | 20429.7 ± 2497.7 | 93.5 ± 22.2 |
|  | MG/AS | 151.8 ± 4.6 | 3568.8 ± 585.3 | 24015.1 ± 1582.1 | 200.1 ± 53.6 |
| CCL2 | MG | 1054.3 ± 42.8 | 63300.0^#^ | 26332.7 ± 2873.7 | 34765.1 ± 5064.8 |
|  | AS | 1836.8 ± 214.6 | 4818.5 ± 316.8 | 63300.0^#^ | 2830.3 ± 421.5 |
|  | MG/AS | 3083.5 ± 284.2 | 51704.8 ± 2682.2 | 63300.0^#^ | 15512.4 ± 3573.3 |
| CXCL8 | MG | 463.0 ± 41.2 | 25400.0^#^ | 25400.0^#^ | 3562.3 ± 931.0 |
|  | AS | 319.0 ± 24.9 | 1777.4 ± 183.5 | 25400.0^#^ | 262.5 ± 85.2 |
|  | MG/AS | 458.5 ± 11.6 | 25400.0^#^ | 25400.0^#^ | 994.7 ± 324.6 |
| GM-CSF | MG | ND | ND | ND | ND |
|  | AS | ND | ND | 505.4 ± 5.3 | ND |
|  | MG/AS | ND | 5.0 ± 0.8 | 383.3 ± 29.4 | ND |
| IL-6 | MG | ND | 2299.6 ± 216.6 | 425.5 ± 110.3 | 48.4 ± 1.9 |
|  | AS | 16.6 ± 1.6 | 78.1 ± 3.1 | 19700.0^#^ | 39.9 ± 9.6 |
|  | MG/AS | 23.9 ± 2.3 | 855.8 ± 210.4 | 19501.8 ± 343.3 | 87.8 ± 26.7 |
| IL-10 | MG | ND | 935.6 ± 73.1 | 13.3 ± 2.4 | ND |
|  | AS | 1.5 ± 0.1 | 1.5 ± 0.2 | 4.4 ± 0.6 | ND |
|  | MG/AS | 4.7 ± 0.5 | 610.0 ± 36.9 | 328.6 ± 29.3 | 3.2 ± 0.8 |
| TNF-α | MG | ND | 230.3 ± 18.2 | N/A | 17.6 ± 3.7 |
|  | AS | ND | ND | N/A | ND |
|  | MG/AS | ND | 105.8 ± 32.0 | N/A | 6.8 ± 9.6 |
| IL-1β | MG | ND | 4.8 ± 0.6 | N/A | 2.2 ± 0.5 |
|  | AS | ND | ND | N/A | ND |
|  | MG/AS | ND | 3.0 ± 0.9 | N/A | ND |

^#^The measured value was above the detection limit and has been set to maximum detectable value of the analyte. ND= not detected. N/A =not applicable.

**Supplementary Table 3.** Inflammatory secretion (mean ± SD) in control and stimulated microglia compartment (MG C), astrocyte compartment (AS C) and microglia–astrocyte coculture compartment (MG/AS C) measured with a multiplex assay (n= 2–3 with 2 technical replicates).

| **Analyte (pg/mL)** | **Culture** | **Ctrl** | **LPS** | **TNF-α/IL-1β** | **IFN-γ** |
| --- | --- | --- | --- | --- | --- |
| CXCL10 | MG C | ND | 3119.9 ± 501.6 | 182.0 ± 98.0 | 5166.1 ± 468.9 |
|  | AS C | ND | 9.5 ± 13.4 | 1830.4 ± 324.6 | 897.7 ± 76.5 |
|  | MG/AS C | ND | 1368.2 ± 497.5 | 2730.2 ± 189.3 | 3581.3 ± 754.1 |
| CXCL5 | MG C | ND | 380.4 ± 38.5 | 75.5 ± 4.4 | ND |
|  | AS C | 7.7 ± 10.9 | 34.0 ± 1.6 | 1774.9 ± 174.8 | ND |
|  | MG/AS C | 7.0 ± 12.1 | 228.3 ± 11.5 | 1950.1 ± 23.8 | 15.2 ± 13.2 |
| CCL2 | MG C | 184.5 ± 21.8 | 12347.9 ± 1756.9 | 6883.7 ± 935.3 | 3709.8 ± 449.4 |
|  | AS C | 279.2 ± 14.6 | 419.4 ± 4.5 | 3743.3 ± 27.9 | 411.0 ± 14.0 |
|  | MG/AS C | 435.8 ± 44.1 | 2665.7 ± 345.1 | 6690.4 ± 425.2 | 950.6 ± 80.1 |
| CXCL8 | MG C | 66.6 ± 5.8 | 25079.6 ± 554.9 | 8584.4 ± 233.4 | 323.3 ± 42.2 |
|  | AS C | 17.9 ± 2.0 | 91.6 ± 8.9 | 16746.2 ± 1139.4 | 48.5 ± 52.0 |
|  | MG/AS C | 45.0 ± 18.5 | 2016.8 ± 302.6 | 22930.2 ± 507.8 | 57.2 ± 14.8 |
| GM-CSF | MG C | ND | ND | ND | ND |
|  | AS C | ND | ND | 78.4 ± 9.3 | ND |
|  | MG/AS C | ND | ND | 80.8 ± 6.7 | ND |
| IL-6 | MG C | ND | 784.2 ± 6.2 | 158.5 ± 20.0 | 14.9 ± 1.3 |
|  | AS C | ND | 19.3 ± 3.2 | 2937.3 ± 119.7 | 14.9 ± 0.6 |
|  | MG/AS C | 7.6 ± 6.9 | 133.8 ± 14.6 | 3802.5 ± 409.8 | 26.2 ± 5.1 |
| IL-10 | MG C | ND | 302.5 ± 66.7 | 3.6 ± 0.8 | ND |
|  | AS C | ND | ND | ND | ND |
|  | MG/AS C | 0.4 ± 0.7 | 24.2 ± 5.9 | 11.4 ± 1.5 | ND |
| TNF-α | MG C | ND | 141.5 ± 12.9 | N/A | ND |
|  | AS C | ND | ND | N/A | ND |
|  | MG/AS C | ND | 3.4 ± 5.9 | N/A | ND |
| IL-1β | MG C | ND | ND | N/A | ND |
|  | AS C | ND | ND | N/A | ND |
|  | MG/AS C | ND | ND | N/A | ND |

ND= not detected; N/A =not applicable.

**Supplementary Table 4.** Fold changes (FC) in cytokine levels between cocultures and microglia and astrocyte monocultures in conventional cultures and microfluidic coculture platform.

|  | **Conventional cultures** | |  | **Microfluidic coculture platform** | |
| --- | --- | --- | --- | --- | --- |
|  | MG/AS vs. MG^1^ | MG/AS vs. AS^2^ |  | MG/AS C vs. MG C^1^ | MG/AS C vs. AS C^2^ |
|  | LPS | TNF-α/IL-1β |  | LPS | TNF-α/IL-1β |
| Cytokine |  |  |  |  |  |
| CXCL10 | **11.8** | 1.04 |  | **0.4** | **1.5** |
| CXCL5 | 0.9 | 1.2 |  | **0.6** | 1.1 |
| CCL2 | **0.8** | 1.0 |  | **0.2** | **1.8** |
| CXCL8 | 1.0 | 1.0 |  | **0.1** | **1.4** |
| GM-CSF | **2.0^#^** | **0.8** |  | ND | 1.0 |
| IL-6 | **0.4** | 1.0 |  | **0.2** | **1.3** |
| IL-10 | **0.7** | **74.7** |  | **0.1** | **11.5**^¤^ |
| TNF-α | **0.5** | N/A |  | **0.02** | N/A |
| IL-1β | **0.6** | N/A |  | ND | N/A |

ND= not detected; N/A =not applicable. Statistically significant values for cytokine concentration comparisons in conventional cultures (Fig. 3 C and D) and microfluidic coculture platform (Fig 6 C and D) are highlighted in bold.

^1^ Calculated by dividing obtained mean concentration of coculture of MG/AS with MG monocultures

^2^ Calculated by dividing obtained mean concentration of coculture with AS cultures

^#^ FC calculated using detection limit (2.5 pg/ml) of the kit for MG monoculture

¤ FC calculated using detection limit (0.99 pg/ml) of the kit for AS C sample
